# Regorafenib monotherapy as second-line treatment of patients with *RAS*-mutant advanced colorectal cancer (STREAM): an academic, multicenter, single-arm, two-stage, phase II study

**DOI:** 10.1016/j.esmoop.2022.100748

**Published:** 2023-01-03

**Authors:** C. Cardone, A. De Stefano, G. Rosati, A. Cassata, L. Silvestro, M. Borrelli, E. Di Gennaro, C. Romano, A. Nappi, N. Zanaletti, F. Foschini, R. Casaretti, F. Tatangelo, S. Lastoria, M. Raddi, D. Bilancia, V. Granata, S. Setola, A. Petrillo, C. Vitagliano, P. Gargiulo, L. Arenare, A. Febbraro, E. Martinelli, F. Ciardiello, P. Delrio, A. Budillon, M.C. Piccirillo, A. Avallone

**Affiliations:** 1 Experimental Clinical Abdominal Oncology Unit, Istituto Nazionale Tumori-IRCCS-Fondazione G. Pascale, Naples, Italy; 2 Medical Oncology Unit, S. Carlo Hospital, Potenza, Italy; 3 Experimental Pharmacology Unit, Istituto Nazionale Tumori-IRCCS-Fondazione G. Pascale, Naples, Italy; 4 Pathology Unit, Istituto Nazionale Tumori-IRCCS-Fondazione G. Pascale, Naples, Italy; 5 Nuclear Medicine Unit, Istituto Nazionale Tumori-IRCCS-Fondazione G. Pascale, Naples, Italy; 6 Radiology Unit, Istituto Nazionale Tumori-IRCCS-Fondazione G. Pascale, Naples, Italy; 7 Clinical Trial Unit, Istituto Nazionale Tumori-IRCCS-Fondazione G. Pascale, Naples, Italy; 8 Hospital Sacro Cuore di Gesu, Fatebenefratelli, Benevento, Italy; 9 Medical Oncology, Precision Medicine Department, University of Campania Luigi Vanvitelli, Naples, Italy; 10 Colorectal Oncological Surgery, Istituto Nazionale Tumori-IRCCS-Fondazione G. Pascale, Naples, Italy

**Keywords:** metastatic colorectal cancer, *RAS* mutant, regorafenib, second-line, [^18^F]-FDG PET/CT

## Abstract

**Background:**

Maintaining angiogenesis inhibition and switching the chemotherapy backbone represent the current second-line therapy in patients with *RAS*-mutant metastatic colorectal cancer (mCRC). Regorafenib, an oral multikinase inhibitor, prolonged overall survival (OS) in the chemorefractory setting.

**Materials and methods:**

STREAM was an academic, multicenter, single-arm phase II trial, evaluating the activity of regorafenib in *RAS*-mutant mCRC, in terms of the rate of patients who were progression-free after 6 months from study entry (6mo-PF). Patients were pretreated with fluoropyrimidine, oxaliplatin, and bevacizumab. According to Simon’s two-stage design, ≥18 patients 6mo-PF were needed in the overall population (*N* = 46). Secondary endpoints were safety, objective response rate (ORR), progression-free survival (PFS), and OS. Early metabolic response by [^18^F]2-fluoro-2-deoxy-D-glucose-positron emission tomography/computed tomography ([^18^F]-FDG PET/CT) scan was an exploratory endpoint. EudraCT Number: 2015-001105-13.

**Results:**

The number of patients 6mo-PF was 8/22 at the first stage and 14/46 in the overall population. The ORR was 10.9%, disease control rate was 54.6%, median (m)PFS was 3.6 months [95% confidence interval (CI) 1.9-6.7 months], mOS was 18.9 months (95% CI 10.3-35.3 months), and mPFS2 (from study entry to subsequent-line progression) was 13.3 months (95% CI 8.4-19.7 months). Long benefiter patients (>6mo-PF) significantly more often had a single metastatic site and lung-limited disease. No unexpected toxicity was reported. Grade ≥3 events occurred in 39.1% of patients, with hand–foot syndrome (13%), fatigue, and hyperbilirubinemia (6.5%) occurring mostly. Baseline metabolic assessment was associated with OS in the multivariate analysis, while early metabolic response was not associated with clinical outcomes.

**Conclusions:**

The study did not meet its primary endpoint. However, regorafenib was well tolerated and did not preclude subsequent treatments. Patients with good prognostic features (single metastatic site and lung-limited disease) reported clinical benefit with regorafenib. The exploratory metabolic analysis suggests that baseline [^18^F]-FDG PET/CT might be useful to select patients with a favorable outcome. A chemotherapy-free interval with regorafenib was associated with durable disease control in a selected group of patients with favorable clinical characteristics.

## Highlights

• Early-lines treatment of patients with *RAS*-mutant mCRC consists of chemotherapy and antiangiogenic agents.
• STREAM evaluated regorafenib as second line for *RAS*-mutant mCRC, in terms of rate of patients progression-free at 6 months.
• The primary endpoint of STREAM was not met. Regorafenib had no unexpected toxicity and did not preclude subsequent therapies.
• A chemotherapy-free interval with regorafenib was beneficial in patients with good prognostic features.
• Baseline [^18^F]-FDG PET/CT might be useful to select patients with favorable outcomes.

## Introduction

Colorectal cancer (CRC) is the third leading cause of mortality in Europe.^1^ Despite substantial improvements in the molecular knowledge of CRC biology and the introduction of novel therapies, the 5-year relative survival rate of patients with metastatic CRC (mCRC) reaches to ∼14%.^2^ It is estimated that ∼50% of patients with mCRC harbor a *RAS* mutation. The RAS protein, a member of the G protein family, is involved in the signal of the mitogen-activated protein kinases signaling pathway.^3^ Activating mutations in *RAS* with aberrant downstream signaling represent a predictive biomarker of resistance to epidermal growth factor receptor inhibition and are associated with more aggressive tumor biology and dismal survival.^4^ Standard treatment in patients with *RAS*-mutant mCRC, not eligible for surgery or local procedures, currently includes chemotherapy (fluoropyrimidines, oxaliplatin, irinotecan) alone or in combination with antiangiogenic agents followed, upon progression, by the sequential use of regorafenib or trifluridine-tipiracil.^5^

The phase III ML18147 and VELOUR trials introduced the biological concept that maintaining angiogenesis inhibition after first-line setting is beneficial for patients with mCRC, providing new second-line treatment options for patients with *RAS*-mutant disease.^6^^,^^7^ Indeed, these trials showed the benefit of aflibercept in combination with FOLFIRI regimen in the second-line setting independently of prior use of bevacizumab, and the efficacy of continuing bevacizumab, switching only chemotherapy, after the failure of a bevacizumab-based first-line treatment, independently of *KRAS* status.^8^ The goals of care in mCRC are primarily intended to prolong survival and maintain the patient’s quality of life (QOL), ensuring a continuum of care. In this regard, maintaining angiogenesis inhibition and switching to a chemotherapy-free strategy by using an oral drug with a different mechanism of action would represent a relevant therapeutic option after first-line therapy. Regorafenib, an oral multikinase inhibitor targeting angiogenesis (i.e. VEGFR1, VEGFR2, VEGFR3, and TIE2), oncogenesis (i.e. KIT, RET, RAF1, BRAF, and BRAFV600E), and tumor microenvironment (PDGFR and FGFR) showed activity in CRC.^9^^,^^10^ In the phase III CORRECT trial, regorafenib demonstrated a survival benefit in chemorefractory patients with an acceptable safety profile.^11^ The phase IIIb CONSIGN study confirmed regorafenib efficacy and safety profile, irrespectively of *RAS* status, reinforcing the importance of dose modifications to manage and prevent adverse events (AEs).^12^ Collectively, these results strengthened the evidence for maintaining angiogenesis inhibition after disease progression, making regorafenib a suitable treatment to be explored even in an earlier line of therapy. On these bases, we conducted a single-arm phase II study of regorafenib monotherapy to test the activity and safety in the second-line treatment of patients with *RAS*-mutant mCRC previously treated with oxaliplatin-based chemotherapy plus bevacizumab. Based on previous findings supporting the use of early metabolic response to predict the efficacy of chemotherapy plus bevacizumab, an exploratory metabolic evaluation with [^18^F]2-fluoro-2-deoxy-D-glucose-positron emission tomography/computed tomography ([^18^F]-FDG PET/CT) scan, 14 days after the start of treatment, has been carried out to identify the patients most likely to benefit from regorafenib therapy.^13^

Correlative translational studies, aimed at a better elucidation of the mechanism of action of regorafenib and the identification of predictive biomarkers, will be described separately.

## Materials and methods

### Study design and inclusion and exclusion criteria

The STREAM trial was an academic, multicenter, single-arm, two-stage, phase II study conducted at four centers in Italy.

Eligible patients had a confirmed diagnosis of *RAS*-mutant mCRC with a disease progression during or following treatment with fluoropyrimidine, oxaliplatin, and bevacizumab, and were not immediately candidate to treatment with irinotecan. Other major inclusion criteria were age ≥18 years; adequate organ functions; Eastern Cooperative Oncology Group (ECOG) performance status of 0 or 1; and at least one measurable lesion, as defined by the Response Evaluation Criteria in Solid Tumors (RECIST) version 1.1.

All patients provided written informed consent. The study was conducted following the principles of the Declaration of Helsinki and the International Conference on Harmonization and Good Clinical Practice guidelines. Ethics Committees of the participating centers approved the study protocol (EudraCT Number: 2015-001105-13; available online as Supplementary Material S1, available at https://doi.org/10.1016/j.esmoop.2022.100748).

### Procedures

Regorafenib was given orally at the approved schedule, 160 mg on days 1-21 in q28 treatment cycles. The study protocol allowed dose reductions and interruptions due to AEs. Two dose reductions were permitted, up to a dose of 80 mg. In case of simultaneous treatment-related AEs leading to different dose modifications, the lowest dose level was indicated as per protocol. If the AEs requiring dose reduction returned to baseline grades after a full cycle of therapy, dose re-escalation was allowed. Guidance on toxicity management is detailed in the protocol (Supplementary Material S1, available at https://doi.org/10.1016/j.esmoop.2022.100748).

Treatment was continued until disease progression, occurrence of an AE requiring treatment cessation, withdrawal of consent, or motivated investigator decision to terminate treatment. Safety was assessed and graded with the Common Terminology Criteria for Adverse Events (CTCAE) version 4.0. Patients who received at least one dose of study drug were considered assessable for safety assessment.

Tumor assessments were carried out in accordance with RECIST version 1.1 by expert radiologists at baseline, within 28 days before initiation of study treatment, at weeks 6 and 12, and every 3 months thereafter, until disease progression.

For exploratory objectives, an [^18^F]-FDG PET/CT scan was carried out within 14 days before initiation of study treatment and after 14 days after drug initiation, to evaluate early metabolic changes.

For each lesion, the following PET-derived parameters have been calculated: Highest maximum standardized uptake value (SUVmax), defined as the highest SUVmax among all evaluable lesions; total SUVmax, defined as the sum of the SUVmax of all evaluable lesions; highest total lesion glycolysis (TLG), defined as the highest TLG among all evaluable lesions. Among these parameters, the SUVmax (the maximum pixel value measured in the visualized lesion) and the TLG) (the average SUVs in the regions of interest × metabolic tumor volume) were evaluated.

### Outcomes

The primary objective was to assess the antitumor activity of regorafenib, measured as the rate of assessable patients alive and not progressed after 6 months from study entry. Events were defined as any of the following occurring within 6 months from registration: progression of disease according to RECIST version 1.1, including deterioration of clinical conditions preventing radiological restaging; death for any cause; interruption of regorafenib due to AEs followed by initiation of an alternative antineoplastic treatment.

Secondary objectives were to describe toxicity, graded according to CTCAE 4.0; objective response rate (ORR), defined as the number of complete plus partial responses divided by the number of patients enrolled; disease control rate (DCR), defined as the number of complete response, partial response and stable disease, divided by the number of patients enrolled; progression-free survival (PFS), defined as the time from registration to progression or death without progression, whichever occurred first; overall survival (OS), defined as the time from registration to the date of death due to any cause; early metabolic response with [^18^F]-FDG PET/CT, defined as a reduction of ≥50% of SUVmax or TLG after 2 weeks of treatment. In addition, PFS2 has been assessed, defined as the PFS time from study entry to the occurrence of progression to the subsequent line of treatment or death without progression, whichever occurred first.

Intra-patient PFS ratios were calculated. Growth modulation index (GMI) was defined as the ratio of the PFS of study treatment with regorafenib to the PFS of the first line of therapy.^14^ Moreover, to account for false-positive and false-negative results, a modified PFS ratio was calculated, converting PFS during first-line treatment <2 months to 2 months, and PFS with study drug >6 months to 24 months.^15^ Both GMI and modified PFS were considered as surrogates of activity if their ratios were >1.33.

Translational exploratory objectives aimed to study the molecular determinants of response and resistance to study treatment and included molecular characterization of solid and liquid biopsies in tumor and blood samples. Genetic alterations found in these exploratory studies, as well as their functional characterization, will be reported separately.

### Statistical analysis

The STREAM study design was developed according to Simon’s two-stage optimal design, to minimize the number of patients exposed to a possibly inactive drug, considering that alternative treatment options existed, although not satisfactory and likely to be more toxic.

Setting type I and II errors at 10% to test the null hypothesis that the rate of patients alive without progression at 6 months was 30% or less versus the alternative hypothesis of 50% or more, 22 assessable patients were required in the first stage. If eight patients were alive without progression after 6 months, a further 24 assessable patients would have been enrolled in the second stage for an overall sample size of 46 assessable patients. The study would have been considered positive if at least 18 patients out of 46 were alive without progression at 6 months.

Differences between categorical data within subgroups were measured using parametrical tests, χ^2^, and Fisher’s exact tests, when adequate. Survival curves were described according to the Kaplan–Meier product-limit method. Patients who received at least one study drug dose were eligible for safety and efficacy analysis. Compliance was evaluated in terms of relative dose intensity (RDI) calculated as the actual dose delivered, defined as the total amount of regorafenib delivered over time, divided by the planned full dose as per protocol over the same time. A univariate Cox proportional hazards model was applied to explore the association between clinical characteristics and OS or PFS. A multivariate model was carried out according to a backward elimination of factors showing a *P* value of <0.10 in the univariate analysis. In all statistical tests, two-sided *P* < 0.05 indicated statistical significance. For each patient and type of toxic effects, the worst degree observed during treatment was used for analysis. Follow-up was completed on 30 December 2021. Statistical analyses were carried out using STATA MP, version 14.1 (StataCorp LLC, College Station, TX).

## Results

### Clinical outcomes

From 11 November 2015, to 12 December 2020, 48 patients were enrolled in four centers. Two patients withdrew consent immediately after study registration and were not included in the analysis. Therefore, 46 patients were assessable, as planned, for efficacy and safety analysis.

Baseline demographics and disease characteristics are summarized in Table 1**.**

**Table 1:** Patients’ characteristics

	Overall population (*N* = 46)	Long benefiters (n = 14)	Poor benefiters (*n* = 32)	*P* value
Age—median (IQR), years	67.0 (56.3-73.1)	67.6 (57.4-71.3)	66.7 (56.1-74.1)	0.848
Sex *n* (%)				
Male	23 (50.0)	6 (42.9)	17 (53.1)	0.522
Female	23 (50.0)	8 (57.1)	15 (46.9)	
ECOG performance status *n* (%)				
0	37 (80.4)	13 (92.9)	24 (75.0)	0.160
1	9 (19.6)	1 (7.1)	8 (25.0)	
Tumor sidedness *n* (%)				
Left	32 (69.6)	9 (64.3)	23 (71.9)	0.607
Right	14 (30.4)	5 (35.7)	9 (28.1)	
Metastatic sites at baseline *n* (%)				
1	15 (32.6)	8 (57.1)	7 (21.9)	**0.019**
>1	31 (67.4)	6 (42.9)	25 (78.1)	
Metastatic sites *n* (%)				
Lung-limited	12 (26.1)	8 (57.1)	4 (12.5)	**0.005**
Lung and other	24 (52.2)	5 (35.7)	19 (59.4)	
Other, no lung	10 (21.7)	1 (7.1)	9 (28.1)	
First-line duration *n* (%)				
>6 months	25 (54.4)	11 (78.6)	14 (43.7)	**0.029**
≤6 months	21 (45.6)	3 (21.4)	18 (56.3)	
Histology *n* (%)				
Adenocarcinoma	40 (87.0)	12 (85.7)	28 (87.5)	0.280
Mucinous	5 (10.9)	1 (7.1)	4 (12.5)	
NA	1 (2.2)	1 (7.1)	0 (0.00)	
Primary tumor surgery *n* (%)				
Yes	40 (87.0)	12 (85.7)	28 (87.5)	0.87
No	6 (13.0)	2 (14.3)	4 (12.5)	
Highest SUVmax (IQR)^a^	12.0 (7.0-15.7)	9.5 (4.0-12.6)	14.0 (7.9-20.6)	0.06
Total SUVmax (IQR)^a^	21.4 (12.5-33.3)	12.5 (7.0-18.7)	26.8 (17.1-38.8)	**0.04**
Highest TLG (IQR)^a^	39.5 (5.7-110.2)	22.4 (5.7-105.0)	46.0 (15.9-110.2)	0.38

Long benefiters (>6 months progression-free); poor benefiters (<6 months progression-free).

Bold values indicate *P* < 0.05.

ECOG, Eastern Cooperative Oncology Group; IQR, interquartile range; NA, not available; SUVmax, maximum standardized uptake value; TLG, total lesion glycolysis.

a Metabolic assessment carried out in 30 patients.

The median age of the study population was 67.0 years; 50.0% (*n* = 23) of the patients were male; 80.4% (*n* = 37) had an ECOG performance status of 0; 69.6% (*n* = 32) had a left-sided tumor location; 32.6% (*n* = 15) had a baseline low burden of disease with the involvement of a single organ as metastatic site; 26.1% (*n* = 12) had a lung-limited disease; 45.6% (*n* = 21) experienced progression to first-line chemotherapy within 6 months of first-line therapy.

All patients, as per inclusion criteria, had *RAS*-mutant disease. Three patients harbored *NRAS* mutations (6.5%), two of whom had a concomitant *KRAS* mutation; in all other cases a mutation in *KRAS* was reported.

The number of assessable patients treated with regorafenib alive and not progressed at 6 months was 8/22 at the first stage of the study, which was passed, while it was 14/46 patients overall, as compared to the 18 required by the study design. Therefore, the study did not meet its primary endpoint.

The overall response rate was 10.9%, and DCR was 54.6%. In details, 25 patients obtained disease control, of which 1 subject achieved a complete response, 4 achieved a partial response, and 20 achieved a stable disease according to RECIST version 1.1 (Table 2).

**Table 2:** Outcomes summary

	Overall population (*N* = 46)	Long benefiters (*n* = 14)	Poor benefiters (*n* = 32)
Median PFS (95% CI), months	3.6 (1.9-6.7)	10.2 (8.6-13.5)	1.9 (1.6-3.5)
Median OS (95% CI), months	18.9 (10.3-35.3)	38.8 (23.9-NR)	13.4 (7.9-18.9)
Disease control rate, *n* (%)	25 (54.3)	14 (100)	11 (34.4)
Response	5 (10.9)	5 (35.7)	0 (0)
Stable disease, *n* (%)	20 (43.5)	9 (64.3)	11 (34.3)
Progressive disease, *n* (%)	21 (45.6)	0 (0)	21 (65.6)
Complete response, *n* (%)	1 (2.2)	1 (7.1)	0 (0)
Partial response, *n* (%)	4 (8.7)	4 (28.6)	0 (0)

Long benefiters (>6 months progression-free); Poor benefiters (<6 months progression-free).

CI, confidence interval; NR, not reached; OS, overall survival; PFS, progression-free survival.

**Figure 1:**
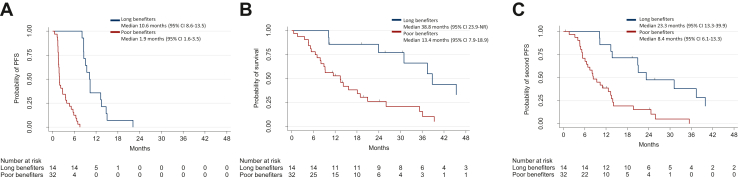
**Kaplan–Meier curves for long and poor benefiters to regorafenib.** (A) Progression-free survival. (B) Overall survival. (C) Progression-free survival 2. Long benefiters (>6 months progression-free); poor benefiters (<6 months progression-free). CI, confidence interval; NR, not reached; PFS, progression-free survival.

The median number of administered cycles was 3.0 [interquartile range (IQR) 2.0-8.0], with a median treatment duration of 2.8 months [95% confidence interval (CI) 1.7-6.2 months]. The RDI was 81.4% (IQR 57.1%-97.3%) (Supplementary Table S1, available at https://doi.org/10.1016/j.esmoop.2022.100748). Thirteen out of 46 patients (28.3%) delayed treatment administration, 27 patients (58.7%) required at least one dose reduction due to the occurrence of treatment-related AEs (Supplementary Table S1, available at https://doi.org/10.1016/j.esmoop.2022.100748). At a median follow-up of 50.2 months (95% CI 24.2-56.3 months), the median PFS was 3.6 months (95% CI 1.9-6.7 months) and median OS was 18.9 months (95% CI 10.3-35.3 months) (Figure 1A and B, Table 2).

At the univariate analysis for median PFS, no clinical characteristic was significantly associated with favorable outcome (Supplementary Table S2, available at https://doi.org/10.1016/j.esmoop.2022.100748).

In the univariate analysis for median OS, the number of metastatic sites and the presence of lung-limited disease were associated with a more favorable outcome. Nevertheless, at the multivariate analysis for OS, only the number of metastatic sites was significantly associated with improved outcomes [hazard ratio (HR) = 3.16, 95% CI 1.30-7.70; *P* = 0.011] (Table 3).

**Table 3:** Univariate and multivariate analysis for overall survival

Covariate	Univariate analysis			Multivariate analysis		
	HR	95% CI	*P* value	HR	95% CI	*P* value
Age	0.99	0.96-1.03	0.728	—	—	—
Sex	1.27	0.64-2.54	0.492	—	—	—
ECOG Performance Status	1.83	0.82-4.09	0.141	—	—	—
Metastatic sites at baseline (>1 versus 1)	3.62	1.54-8.53	**0.003**	3.16	1.30-7.70	**0.011**
Tumor sidedness	0.81	0.36-1.80	0.604	—	—	—
Lung-limited disease	0.33	0.14-0.80	**0.013**	0.41	0.16-1.02	0.054

Bold value indicates *P* < 0.05.

CI, confidence interval; ECOG, Eastern Cooperative Oncology Group; HR, hazard ratio.

### Safety

At least one treatment-related AE (any grade) occurred in 43 patients (91.5%). The most common AEs were hand–foot skin reactions (*n* = 18, 38.3%), fatigue (*n* = 15, 31.9%), increase in bilirubin (*n* = 12, 25.5%), decrease in platelet count (*n* = 9, 19.1%), anemia (*n* = 8, 17.0%), maculopapular rash (*n* = 8, 17.0%), voice alteration (*n* = 7, 14.9%), mucositis (*n* = 7, 14.9%), fever (*n* = 6, 12.8%), diarrhea (*n* = 5, 10.6%), increase in aspartate aminotransferase (*n* = 4, 8.5%), hypertension (*n* = 4, 8,5%), increase in alanine aminotransferase (*n* = 3, 6.4%), and decrease in neutrophil count (*n* = 3, 6.4%).

Grade 3 or higher treatment-related AEs occurred in 18 patients (38.3%). The most common grade ≥3 AEs were hand–foot skin reaction (*n* = 6, 12.8%) fatigue (*n* = 3, 6.4%), increase in blood bilirubin (*n* = 3, 6.4%), maculopapular rash (*n* = 2, 4.3%), and mucositis (*n* = 2, 4.3%). No unexpected toxic effect was observed. A detailed toxicity profile is reported in Supplementary Table S3, available at https://doi.org/10.1016/j.esmoop.2022.100748. AEs occurring in at least 5% of patients and grade ≥3 AEs are reported in Table 4**.**

**Table 4:** Treatment-related adverse events occurring in at least 5% of patients and grade ≥3 adverse events

Adverse event	Any grade *N*	%	Grade≥3 *N*	%
Hand–foot syndrome	18	38.3	6	12.8
Fatigue	15	31.9	3	6.4
Blood bilirubin increased	12	25.5	3	6.4
Platelet count decreased	9	19.1	0	0.0
Anemia	8	17.0	0	0.0
Maculopapular rash	8	17.0	2	4.3
Oral mucositis	7	14.9	2	4.3
Voice alteration	7	14.9	0	0.0
Fever	6	12.8	0	0.0
Diarrhea	5	10.6	0	0.0
Aspartate aminotransferase increased	4	8.5	1	2.1
Hypertension	4	8.5	1	2.1
Alanine aminotransferase increased	3	6.4	0	0.0
Neutrophil count decreased	3	6.4	0	0.0

### Exploratory metabolic data

A total of 30 patients out of 46 underwent a metabolic assessment. At the baseline evaluation, the median highest SUVmax was 12.0 (IQR 7.0-15.7), the median total SUVmax was 21.4 (IQR 12.5-33.3), and the median highest TLG was 39.5 (IQR 5.7-110.2) (Table 1).

After 2 weeks of treatment, three patients (10.0%) had a metabolic response according to the highest SUVmax, five (16.7%) according to total SUVmax and four (13.3%) according to the highest TLG. The early metabolic response was not associated with clinical outcomes (Supplementary Table S4, available at https://doi.org/10.1016/j.esmoop.2022.100748).

Baseline metabolic assessment was not associated with PFS (Supplementary Table S5, available at https://doi.org/10.1016/j.esmoop.2022.100748).

Conversely, baseline metabolic evaluation, assessed with the highest SUVmax and total SUVmax, was associated with OS. As per univariate analysis, carried out in the group of 30 patients undergoing metabolic assessment, a numerical increased value of baseline highest SUVmax (HR 1.10, 95% CI 1.03-1.17, *P* = 0.003) and baseline total SUVmax values (HR 1.04, 95% CI 1.02-1.05, *P* < 0.001) was significantly associated with worse OS, whereas no association was found with the highest TLG. In addition, the presence of lung-limited disease (HR 0.15, 95% CI 0.03-0.67, *P* = 0.01) and the number of metastatic sites (HR 5.92, 95% CI 1.36-25.8, *P* = 0.02) were significantly associated with OS (Supplementary Table S6, available at https://doi.org/10.1016/j.esmoop.2022.100748).

Moreover, in a multivariate model including the number of metastatic sites, the presence of lung-limited disease, and total SUVmax, the only covariate significantly associated with survival was baseline metabolic evaluation as total SUVmax (HR 1.03, 95% CI 1.01-1.05, *P* = 0.007). Of note, the total SUVmax was the only metabolic covariate included in the multivariate model due to the functional overlapping with the highest SUVmax and due to its better representation of the metabolic activity (Supplementary Table S6, available at https://doi.org/10.1016/j.esmoop.2022.100748).

### Clinical characterization of long benefiters

A subgroup of 14 patients, hereinafter referred to as long benefiters, experienced a favorable outcome with regorafenib, being on treatment for >6 months. Conversely, 32 patients, defined as poor benefiters, were on treatment for <6 months.

According to baseline characteristics, long benefiters most frequently presented with a single metastatic site (*P* = 0.019) and with a lung-limited disease (*P* = 0.005) (Table 1). Long benefiters to regorafenib have had a favorable outcome in the first-line setting also, with a lower rate of early progression to first-line treatment, as compared to poor benefiters (first-line PFS ≤ 6 months, *n* = 3, 21.4% versus first-line PFS > 6 months, *n* = 18, 56.3%; *P* = 0.029) (Table 1). Long benefiters reported a favorable median PFS of 10.2 months (95% CI 8.6-13.5 months) and median OS of 38.8 months [95% CI 23.9-not reached (NR)], as compared to poor benefiters, who reported a median PFS of 1.9 months (95% CI 1.6-3.5 months) and a median OS of 13.4 months (95% CI 7.9-18.9 months). Long benefiters had an ORR of 35.7%, whereas no response was observed in poor benefiters who obtained a DCR of 34.4% (Table 2).

The median treatment duration was 9.0 months (95% CI 8.0-10.2 months) for long benefiters and 1.7 months (95% CI 1.4-2.5 months) for poor benefiters, corresponding to a total of nine cycles (IQR 8-10) versus two cycles (IQR 2-3), respectively. However, no difference in treatment delay and RDI was reported among the two groups (Supplementary Table S1, available at https://doi.org/10.1016/j.esmoop.2022.100748).

At study treatment discontinuation, third-line chemotherapy was given to a total of 37 patients (80.4%), of which 10 were long benefiters (71%) and 27 were poor benefiters (84%) (Supplementary Table S7, available at https://doi.org/10.1016/j.esmoop.2022.100748).

The median PFS at subsequent line of therapy (mPFS2), calculated from study entry to progression to a subsequent line of treatment, or death, whichever occurred first, was 13.3 months (95% CI 8.4-19.7 months) in the overall population, and was longer in long benefiters with 23.3 months (95% CI 13.3-39.9 months) as compared to poor responders with 8.4 months (95% CI 6.1-13.3 months). Of note, PFS2 calculation included also patients who did not start a subsequent line due to death or clinical deterioration (*n* = 4) (Figure 1C).

At the baseline metabolic assessment, significantly lower median total SUVmax values were observed in long benefiters 12.5 (IQR 7.0-18.7) as compared to poor benefiters 26.8 (IQR 17.1-38.8), *P* = 0.04; a difference, even if not statistically significant, was observed between the two groups for median highest SUVmax values—9.5 (IQR 4.0-12.6) versus 14.0 (IQR 7.9-20.6), *P* = 0.06—whereas no difference was observed in the highest TLG values—39.5 (IQR 5.7-105.0) versus 46.0 (IQR 15.9-110.2), *P* = 0.38 (Table 1).

No difference was observed between the two groups with respect to early metabolic response (Supplementary Table S4, available at https://doi.org/10.1016/j.esmoop.2022.100748).

As a measure of regorafenib activity, GMI and modified PFS ratio were calculated in long benefiters. The median GMI in long benefiters was 0.89 (IQ 0.57-1.93) with 43% of patients (*n* = 6) reporting a GMI > 1.33. The modified PFS ratio, calculated to account for false-negative and false-positive results, was 2.37 (IQ 1.49-2.91), with 79% of patients (*n* = 11) showing a modified PFS ratio > 1.33. As expected, all poor benefiters had a GMI < 1.33.

## Discussion

The STREAM study sought at evaluating regorafenib as a second-line therapy of *RAS*-mutant mCRC. The study did not meet the primary endpoint. However, the results lead to potentially relevant clinical implications.

Firstly, despite the short median PFS observed in the STREAM study, an encouraging OS of 18.9 months was reported, especially if compared to clinical outcomes reported in *RAS*-mutant patients treated within ML18147 and VELOUR trials, where the addition of bevacizumab or aflibercept, respectively, in *RAS*-mutant patients determined an mPFS of 5.5 and 6.5 months and an mOS of 10.4 and 12.6 months, respectively. In the same studies, *RAS*-wild-type patients reached an mPFS of 6.4 and 7.4 months and an mOS of 15.4 and 16.0 months, respectively.^6^^,^^14^^,^^15^, ^16^, ^17^, ^18^

Moreover, despite the short PFS, regorafenib did not impair subsequent treatment outcomes. Overall, 37 patients (80.4%) received a third-line therapy, yielding a favorable median PFS2 of 13.3 months. In line with these results, preclinical data have suggested that regorafenib may play a role in chemosensitization. The observed synergy involves the proapoptotic protein PUMA and the modulation of drug transporters such as ABCB1, affecting the concentration of cytotoxic drugs in the tumor-associated microenvironment.^18^^,^^19^ Moreover, observational data report that in patients treated with chemotherapy followed by regorafenib, subsequent re-treatment with chemotherapy may lead to a response.^20^^,^^21^

Collectively, this evidence might not completely exclude the use of regorafenib in an earlier setting in patients with *RAS*-mutant colorectal cancer.

Secondly, as reported in previous studies, heterogeneous responses to regorafenib have been observed and have to be discussed. Patients who reported limited benefit are characterized by an overall worse prognosis, being associated with an unfavorable outcome across all lines of treatment, irrespective of regorafenib treatment. Indeed, patients with mCRC who experience early progression during first-line chemotherapy showed poor clinical outcomes in the second-line setting regardless of the use of antiangiogenic agents.^22^

Parallelly, the STREAM study showed that there is a subgroup of *RAS*-mutant mCRC patients with considerably favorable outcomes with regorafenib. In line with previous observations, patients with lower metastatic burden and lung-limited disease experienced a longer disease control with regorafenib.^23^, ^24^, ^25^

It is worthwhile to consider that long responder patients were associated with an overall more favorable prognosis, irrespective of the study drug, as observed by the outcomes reported in the first-line setting and in the subsequent lines of treatment. This population might benefit from a personalized approach, with less intensive treatment, in the context of a continuum-of-care strategy devoted to the maintenance of QOL and the prolongation of survival. Nevertheless, as demonstrated by GMI and by modified PFS ratio, an intrinsic effect of regorafenib was observed in long benefiter patients as well.

Thirdly, treatment with regorafenib in the second-line setting was not associated with unexpected toxicities. Recent evidence from the ReDOS and CORRELATE studies suggest that dose optimization by using a dose-escalation strategy is noninferior from an efficacy standpoint, being associated to a decreased rate of toxicity within the first cycles and improving patients’ QOL.^25^^,^^26^

The STREAM study adopted a standard dosing schedule approach since it was developed before the dose-escalation evidences were available. However, the use of preemptive supportive measures, especially with regard to hand–foot syndrome reactions, and appropriate dose modifications were critical for patients’ compliance and treatment management. Therefore, early dose escalation and preemptive supportive measures should be routinely used during regorafenib therapy.

The STREAM study also explored the role of [^18^F]-FDG PET/CT scan as an early imaging biomarker of regorafenib activity. Acknowledging the limited number of patients, the [^18^F]-FDG PET/CT scan at 14 days after study initiation was not predictive of regorafenib activity and clinical outcome. In line with our results, the JACCRO CC-12 study failed to demonstrate the usefulness of FDG PET as an early imaging biomarker of regorafenib in patients with mCRC and supported the use of additional metabolic parameters such as metabolic tumor volume and TLG.^27^

Indeed, baseline metabolic assessment, by using the highest or total SUVmax, might contribute to identifying patients with better prognosis and is more likely to benefit from less intensive treatment.

Within the limitation of the small number of patients included in the metabolic assessment, the STREAM study suggests that the use of baseline [^18^F]-FDG PET/CT might be useful to select patients with favorable OS.

Treatment of *RAS*-mutant CRC is challenged by poor prognosis and limited therapeutic options.

A recent meta-analysis from 14 randomized trials reported that the survival benefit in the second-line setting with chemotherapy alone or in combination with antiangiogenic agents was significantly higher in patients with *RAS*-wild-type mCRC tumors as compared to *RAS*-mutant.^17^

Despite several investigational efforts in the past decades, *RAS* has historically been regarded as an ‘undruggable’ gene.^28^ However, since the STREAM study was conducted, direct *RAS* inhibition has emerged as a possible therapeutic option in several solid malignancies, including CRC. Based on robust preclinical data, several KRAS^G12C^ inhibitors are currently under clinical development, alone or in combination with anti-epidermal growth factor receptor drugs or checkpoint inhibitors, to enhance treatment activity.^29^, ^30^, ^31^ In particular, AMG510 (sotorasib) and MRTX849 (adagrasib) have recently shown encouraging preliminary results in combination with anti-epidermal growth factor receptor agents.^32^^,^^33^ Furthermore, based on the known GTPase activity, KRAS^G12D^ represents a promising drug target, currently being investigated in early clinical trials.^31^

Despite recent advancements, developing novel therapeutic strategies for *RAS*-mutant CRC remains an unmet clinical need. Moreover, improving patients’ selection might allow the optimization of the available treatment options.

### Conclusions

The study did not meet its primary endpoint. However, regorafenib was well tolerated and did not preclude subsequent treatments. Patients with good prognostic features (single metastatic site and lung-limited disease) reported clinical benefit with regorafenib.

The exploratory metabolic analysis suggests that baseline [^18^F]-FDG PET/CT might be useful to select patients with favorable OS.

The identification of patients suitable for a less intensive treatment might guide further development of regorafenib in an earlier setting, prolonging the chemotherapy-free interval within the sequence of treatments for patients with *RAS*-mutant mCRC.
