# Supplementary material for: Regorafenib monotherapy as second-line treatment of patients with RAS-mutant advanced colorectal cancer (STREAM): an academic, multicenter, single-arm, two-stage, phase II study
Source: ESMO Open. 2023 Jan 3;8(1):100748. doi: 10.1016/j.esmoop.2022.100748 (PMC10024144; doi:10.1016/j.esmoop.2022.100748)
Supplement: Supplementary Material [file mmc1.docx]

**Supplementary Contents**

| **Supplementary Table S1** | Treatment administration, Relative dose intensity, Treatment delay and interruption | p.3 |
| --- | --- | --- |
| **Supplementary Table S2** | Univariate and multivariate analysis for Progression Free Survival | p.4 |
| **Supplementary Table S3** | Adverse Events, complete table | p.5 |
| **Supplementary Table S4** | Correlation between early metabolic response and long and poor benefiters | p.7 |
| **Supplementary Table S5** | Baseline metabolic assessment: Univariate analysis and multivariate analysis for Progression Free Survival | p.8 |
| **Supplementary Table S6** | Baseline metabolic assessment: Univariate analysis and multivariate analysis for Overall Survival | p.9 |
| **Supplementary Table S7** | Subsequent treatment lines | p.10 |

**Supplementary Table S1 Treatment administration, Relative dose intensity, Treatment delay and interruption**

|  | Overall population  (N=46) | | Long Benefiters  (N=14) | | Poor Benefiters  (N=32) | |
| --- | --- | --- | --- | --- | --- | --- |
| Time on treatment, mo (95%CI) | 2.8 | (1.7-6.2) | 9.0 | (8.0-10.1) | 1.7 | (1.4-2.5) |
| Cycles, no (IQR) | 3 | (2-8) | 9 | (8-10) | 2 | (2-3) |
| RDI, % (IQR) | 81.4 | (57.1-97.3) | 82.0 | (55.1-95.0) | 81.1 | (57.1-99.2) |
| Treatment delay, n. (%)  No  Yes | 33  13 | 71.7  28.3 | 9  5 | 64.3  35.7 | 24  8 | 75.0  25.0 |
| Dose Reductions, n. (%)  No  Yes | 19  27 | 41.3  58.7 | 6  8 | 42.9  57.1 | 13  19 | 40.6  59.4 |
| mo= Months; RDI=Relative Dose Intensity; IQR=Interquartile Range; Long benefiters (>6 months progression-free); Poor benefiters (<6 months progression-free) | | | | | | |

**Supplementary Table S2 Univariate and multivariate analysis for Progression Free Survival**

| Covariate | Univariate PFS | | | Multivariate PFS | | |  |
| --- | --- | --- | --- | --- | --- | --- | --- |
|  | **HR** | **CI95%** | **P value** | **HR** | **CI 95%** | **P value** |  |
| Age | 0.99 | 0.96-1.02 | 0.521 | - | - | - |  |
| Gender | 1.03 | 0.56-1.87 | 0.924 | - | - | - |  |
| ECOG Performance Status | 1.92 | 0.90- 4.09 | 0.092 | 2.06 | 0.96-4.43 | 0.06 |  |
| Metastatic sites at baseline (>1 vs 1) | 1.58 | 0.85-2.97 | 0.151 | - | - | - |  |
| Tumor sidedness | 0.85 | 0.44-1.62 | 0.623 | - | - | - |  |
| Lung-limited disease | 0.52 | 0.26-1.02 | 0.058 | 0.50 | 0.25-0.98 | **0.04** |  |
| PFS=Progression Free Survival; HR=Hazard Ratio; CI95%=Confidence Interval; ECOG = Easter Cooperative Oncology Group | | | | | | | |

**Supplementary Table S3. All treatment related adverse events**

| **Adverse Event** | **Any grade**  **N. %** | | **G≧3**  **N. %** | | **G1**  **N. %** | | **G2**  **N. %** | | **G3**  **N. %** | | **G4**  **N. %** | | **G5**  **N. %** | |
| --- | --- | --- | --- | --- | --- | --- | --- | --- | --- | --- | --- | --- | --- | --- |
| Total | 43 | 91.5 | 18.0 | 38.3 | 5 | 10.9 | 20 | 43.5 | 16 | 34.8 | 2 | 4.3 | 0 | 0.0 |
| Hand Foot syndrome | 18 | 38.3 | 6.0 | 12.8 | 7 | 15.2 | 5 | 10.9 | 6 | 13.0 | 0 | 0.0 | 0 | 0.0 |
| Fatigue | 15 | 31.9 | 3.0 | 6.4 | 5 | 10.9 | 7 | 15.2 | 3 | 6.5 | 0 | 0.0 | 0 | 0.0 |
| Blood bilirubin increased | 12 | 25.5 | 3.0 | 6.4 | 6 | 13.0 | 3 | 6.5 | 3 | 6.5 | 0 | 0.0 | 0 | 0.0 |
| Platelet count decreased | 9 | 19.1 | 0.0 | 0.0 | 5 | 10.9 | 4 | 8.7 | 0 | 0.0 | 0 | 0.0 | 0 | 0.0 |
| Anemia | 8 | 17.0 | 0.0 | 0.0 | 6 | 13.0 | 2 | 4.3 | 0 | 0.0 | 0 | 0.0 | 0 | 0.0 |
| Rash maculo-papular | 8 | 17.0 | 2.0 | 4.3 | 3 | 6.5 | 3 | 6.5 | 2 | 4.3 | 0 | 0.0 | 0 | 0.0 |
| Mucositis oral | 7 | 14.9 | 2.0 | 4.3 | 5 | 10.9 | 0 | 0.0 | 2 | 4.3 | 0 | 0.0 | 0 | 0.0 |
| Voice alteration | 7 | 14.9 | 0.0 | 0.0 | 7 | 15.2 | 0 | 0.0 | 0 | 0.0 | 0 | 0.0 | 0 | 0.0 |
| Fever | 6 | 12.8 | 0.0 | 0.0 | 4 | 8.7 | 2 | 4.3 | 0 | 0.0 | 0 | 0.0 | 0 | 0.0 |
| Diarrhea | 5 | 10.6 | 0.0 | 0.0 | 1 | 2.2 | 4 | 8.7 | 0 | 0.0 | 0 | 0.0 | 0 | 0.0 |
| Aspartate aminotransferase increased | 4 | 8.5 | 1.0 | 2.1 | 2 | 4.3 | 1 | 2.2 | 1 | 2.2 | 0 | 0.0 | 0 | 0.0 |
| Hypertension | 4 | 8.5 | 1.0 | 2.1 | 3 | 6.5 | 0 | 0.0 | 1 | 2.2 | 0 | 0.0 | 0 | 0.0 |
| Alanine aminotransferase increased | 3 | 6.4 | 0.0 | 0.0 | 2 | 4.3 | 1 | 2.2 | 0 | 0.0 | 0 | 0.0 | 0 | 0.0 |
| Neutrophil count decreased | 3 | 6.4 | 0.0 | 0.0 | 2 | 4.3 | 1 | 2.2 | 0 | 0.0 | 0 | 0.0 | 0 | 0.0 |
| Abdominal pain | 2 | 4.3 | 0.0 | 0.0 | 0 | 0.0 | 2 | 4.3 | 0 | 0.0 | 0 | 0.0 | 0 | 0.0 |
| Alkaline phosphatase increased | 2 | 4.3 | 0.0 | 0.0 | 2 | 4.3 | 0 | 0.0 | 0 | 0.0 | 0 | 0.0 | 0 | 0.0 |
| White blood cell decreased | 2 | 4.3 | 0.0 | 0.0 | 2 | 4.3 | 0 | 0.0 | 0 | 0.0 | 0 | 0.0 | 0 | 0.0 |
| Anorexia | 2 | 4.3 | 0.0 | 0.0 | 1 | 2.2 | 1 | 2.2 | 0 | 0.0 | 0 | 0.0 | 0 | 0.0 |
| Atrial fibrillation | 1 | 2.1 | 1.0 | 2.1 | 0 | 0.0 | 0 | 0.0 | 1 | 2.2 | 0 | 0.0 | 0 | 0.0 |
| Hypothyroidism | 1 | 2.1 | 0.0 | 0.0 | 0 | 0.0 | 1 | 2.2 | 0 | 0.0 | 0 | 0.0 | 0 | 0.0 |
| Colonic perforation | 1 | 2.1 | 1.0 | 2.1 | 0 | 0.0 | 0 | 0.0 | 0 | 0.0 | 1 | 2.2 | 0 | 0.0 |
| Dry mouth | 1 | 2.1 | 0.0 | 0.0 | 1 | 2.2 | 0 | 0.0 | 0 | 0.0 | 0 | 0.0 | 0 | 0.0 |
| Gastritis | 1 | 2.1 | 0.0 | 0.0 | 0 | 0.0 | 1 | 2.2 | 0 | 0.0 | 0 | 0.0 | 0 | 0.0 |
| Gastrointestinal pain | 1 | 2.1 | 0.0 | 0.0 | 1 | 2.2 | 0 | 0.0 | 0 | 0.0 | 0 | 0.0 | 0 | 0.0 |
| Vomiting | 1 | 2.1 | 1.0 | 2.1 | 0 | 0.0 | 0 | 0.0 | 0 | 0.0 | 1 | 2.2 | 0 | 0.0 |
| Laryngitis | 1 | 2.1 | 0.0 | 0.0 | 0 | 0.0 | 1 | 2.2 | 0 | 0.0 | 0 | 0.0 | 0 | 0.0 |
| Urinary tract infection | 1 | 2.1 | 0.0 | 0.0 | 0 | 0.0 | 1 | 2.2 | 0 | 0.0 | 0 | 0.0 | 0 | 0.0 |
| CPK increased | 1 | 2.1 | 0.0 | 0.0 | 0 | 0.0 | 1 | 2.2 | 0 | 0.0 | 0 | 0.0 | 0 | 0.0 |
| Creatinine increased | 1 | 2.1 | 0.0 | 0.0 | 1 | 2.2 | 0 | 0.0 | 0 | 0.0 | 0 | 0.0 | 0 | 0.0 |
| GGT increased | 1 | 2.1 | 0.0 | 0.0 | 1 | 2.2 | 0 | 0.0 | 0 | 0.0 | 0 | 0.0 | 0 | 0.0 |
| Serum amylase increased | 1 | 2.1 | 0.0 | 0.0 | 1 | 2.2 | 0 | 0.0 | 0 | 0.0 | 0 | 0.0 | 0 | 0.0 |
| Hypomagnesemia | 1 | 2.1 | 0.0 | 0.0 | 1 | 2.2 | 0 | 0.0 | 0 | 0.0 | 0 | 0.0 | 0 | 0.0 |
| Hyponatremia | 1 | 2.1 | 1.0 | 2.1 | 0 | 0.0 | 0 | 0.0 | 1 | 2.2 | 0 | 0.0 | 0 | 0.0 |
| Dysgeusia | 1 | 2.1 | 0.0 | 0.0 | 1 | 2.2 | 0 | 0.0 | 0 | 0.0 | 0 | 0.0 | 0 | 0.0 |
| Peripheral motor neuropathy | 1 | 2.1 | 1.0 | 2.1 | 0 | 0.0 | 0 | 0.0 | 1 | 2.2 | 0 | 0.0 | 0 | 0.0 |
| Pneumothorax | 1 | 2.1 | 0.0 | 0.0 | 0 | 0.0 | 1 | 2.2 | 0 | 0.0 | 0 | 0.0 | 0 | 0.0 |
| Alopecia | 1 | 2.1 | 0.0 | 0.0 | 1 | 2.2 | 0 | 0.0 | 0 | 0.0 | 0 | 0.0 | 0 | 0.0 |
| Dry skin | 1 | 2.1 | 0.0 | 0.0 | 1 | 2.2 | 0 | 0.0 | 0 | 0.0 | 0 | 0.0 | 0 | 0.0 |
| Pruritus | 1 | 2.1 | 0.0 | 0.0 | 0 | 0.0 | 1 | 2.2 | 0 | 0.0 | 0 | 0.0 | 0 | 0.0 |
| Skin induration | 1 | 2.1 | 0.0 | 0.0 | 1 | 2.2 | 0 | 0.0 | 0 | 0.0 | 0 | 0.0 | 0 | 0.0 |

**Supplementary Table S4 Correlation between early metabolic response and long and poor responders**

| Early Metabolic Response | Overall population  (N=30) | | | Long Benefiters  (N=9) | | Poor Benefiters  (N=21) | | P value |  |
| --- | --- | --- | --- | --- | --- | --- | --- | --- | --- |
| Highest SUVmax |  |  | |  |  |  |  |  |  |
| No, n. (%) | 27 | | 90.0 | 8 | 88.9 | 19 | 90.5 | 0.894 |  |
| Yes, n. (%) | 3 | | 10.0 | 1 | 11.1 | 2 | 9.5 |  |  |
|  |  | |  |  |  |  |  |  |  |
| Total SUVmax |  | |  |  |  |  |  |  |  |
| No, n. (%) | 25 | | 83.3 | 8 | 88.9 | 17 | 80.9 | 0.593 |  |
| Yes, n. (%) | 5 | | 16.7 | 1 | 11.1 | 4 | 19.1 |  |  |
|  |  | |  |  |  |  |  |  |  |
| Highest TLG |  | |  |  |  |  |  |  |  |
| No, n. (%) | 26 | | 88.9 | 8 | 85.7 | 18 | 87.7 | 0.815 |  |
| Yes, n. (%) | 4 | | 13.3 | 1 | 11.1 | 3 | 14.3 |  |  |
| SUV= Standardized Uptake Value; TLG=Total Lesion Glycolysis; Long benefiters (>6 months progression-free); Poor benefiters (<6 months progression-free) | | | | | | | | |  |

**Supplementary Table S5. Baseline metabolic assessment: Univariate analysis and multivariate analysis for Progression Free Survival**

| Covariate | Univariate PFS | | | Multivariate PFS | | |
| --- | --- | --- | --- | --- | --- | --- |
|  | **HR** | **CI95%** | **P value** | **HR** | **CI95%** | **P value** |
| Total SUVmax ^§^ | 1.01 | 1.00-1.03 | 0.08 | 1.01 | 0.99-1.02 | 0.17 |
| Highest SUVmax ^§^ | 1.06 | 1.00-1.12 | 0.07 |  |  |  |
| Highest TLG | 1.00 | 1.00-1.01 | 0.30 |  |  |  |
| Age | 0.98 | 0.94-1.02 | 0.29 |  |  |  |
| Gender | 1.15 | 0.55-2.43 | 0.71 |  |  |  |
| ECOG Performance Status | 1.48 | 0.59-3.72 | 0.40 |  |  |  |
| Metastatic sites at baseline (>1 vs 1) | 1.97 | 0.78-4.93 | 0.15 |  |  |  |
| Tumor sidedness | 0.70 | 0.28-1.74 | 0.44 |  |  |  |
| Lung-limited disease | 0.42 | 0.17-1.00 | 0.05 | 0.46 | 0.19-1.11 | 0.08 |
| OS=Overall Survival; HR=Hazard Ratio; CI95%=Confidence Interval; ECOG = Easter Cooperative Oncology Group; SUV= Standardized Uptake Value; TLG=Total Lesion Glycolysis | | | | | | |

§Only Total SUVmax was included in the multivariate model due to the functional overlapping with Highest SUVmax, and due to its better representation of the metabolic activity.

**Supplementary Table S6 : Univariate and Multivariate analysis for Overall Survival according to baseline metabolic parameters**

| Covariate | Univariate OS | | | | Multivariate OS | | |
| --- | --- | --- | --- | --- | --- | --- | --- |
|  | **HR** | **CI95%** | **P value** | **HR** | | **CI95%** | **P value** |
| Total SUVmax ^§^ | 1.04 | 1.02-1.05 | **<0.001** | 1.03 | | 1.01-1.05 | **0.007** |
| Highest SUVmax ^§^ | 1.10 | 1.03-1.17 | **0.003** | - | | - | - |
| Highest TLG | 1.00 | 1.00-1.00 | 0.18 | - | | - | - |
| Age | 0.98 | 0.95-1.02 | 0.42 | - | | - | - |
| Gender | 1.28 | 0.55-2.98 | 0.57 | - | | - | - |
| ECOG Performance Status | 1.20 | 0.44-3.25 | 0.72 | - | | - | - |
| Metastatic sites at baseline (>1 vs 1) | 5.92 | 1.36-25.8 | **0.02** | 2.78 | | 0.58-13.3 | 0.20 |
| Tumor sidedness | 0.47 | 0.14-1.59 | 0.22 | - | | - | - |
| Lung-limited disease | 0.15 | 0.03-0.67 | **0.01** | 0.32 | | 0.06-1.56 | 0.16 |
| OS=Overall Survival; HR=Hazard Ratio; CI95%=Confidence Interval; ECOG = Easter Cooperative Oncology Group; SUV= Standardized Uptake Value; TLG=Total Lesion Glycolysis | | | | | | | |

§ Only Total SUVmax was included in the multivariate model due to the functional overlapping with Highest SUVmax, and due to its better representation of the metabolic activity.

**Supplementary Table S7. Subsequent treatment lines**

| Subsequent treatment lines | Overall population  (N=46) | | Long Benefiters  (N=14) | | Poor Benefiters  (N=32) | |  |
| --- | --- | --- | --- | --- | --- | --- | --- |
| Third line |  |  |  |  |  |  |  |
| No treatment, n. (%) | 9 | 19.6 | 4 | 28.6 | 5 | 15.6 |  |
| FOLFIRI + bevacizumab, n. (%) | 13 | 28.3 | 6 | 42.9 | 7 | 21.9 |  |
| FOLFIRI + aflibercept, n. (%) | 20 | 28.3 | 3 | 21.4 | 17 | 53.1 |  |
| Regorafenib beyond PD, n. (%) | 2 | 4.3 | 1 | 7.1 | 1 | 3.1 |  |
| Other, n. (%) | 2 | 4.3 | 0 | 0.0 | 2 | 6.2 |  |
| Fourth line |  |  |  |  |  |  |  |
| No treatment, n. (%) | 30 | 65.2 | 11 | 78.6 | 19 | 59.4 |  |
| Trifluridine-tipiracile, n. (%) | 12 | 26.1 | 2 | 14.3 | 10 | 31.2 |  |
| FOLFOX, n. (%) | 2 | 4.3 | 0 | 0.0 | 2 | 6.3 |  |
| Other, n. (%) | 2 | 4.3 | 1 | 7.1 | 1 | 3.1 |  |
| Fifth line |  |  |  |  |  |  |  |
| No treatment, n. (%) | 42 | 91.3 | 12 | 85.7 | 30 | 93.7 |  |
| Trifluridine-tipiracile, n. (%) | 1 | 2.2 | 1 | 7.14 | 0 | 0.0 |  |
| FOLFOX, n. (%) | 2 | 4.3 | 0 | 0.0 | 2 | 6.3 |  |
| Other, n. (%) | 1 | 2.1 | 1 | 7.14 | 0 | 0.0 |  |
| PD= progressive disease; Long benefiters (>6 months progression-free); Poor benefiters (<6 months progression-free) | | | | | | | |
