# Supplementary material for: Regorafenib monotherapy as second-line treatment of patients with RAS-mutant advanced colorectal cancer (STREAM): an academic, multicenter, single-arm, two-stage, phase II study
Source: ESMO Open. 2023 Jan 3;8(1):100748. doi: 10.1016/j.esmoop.2022.100748 (PMC10024144; doi:10.1016/j.esmoop.2022.100748)
Supplement: Supplementary Table [file mmc2.pdf]

EudraCT Number: 2015-001105-13

Regorafenib monotherapy as second-line treatment of patients with *RAS*-mutant advanced colorectal cancer: a multicentre, single-arm, two-stage, phase 2 study.

Nickname: STREAM (**S**econd-line **T**reatment with **RE**gorafenib in **A**dvanced RAS-**M**utant colorectal cancer)

No-profit promoter: National Cancer Institute, Napoli (NCI)

Principal Investigators

A. Avallone - Abdominal Medical Oncology, NCI
A. Budillon - Experimental Pharmacology, NCI
F. Perrone - Clinical Trials Unit, NCI

Steering committee:

P. Delrio, G. Romano	Abdominal Surgical Oncology, NCI
B. Pecori, P. Muto	Radiotherapy, NCI
V.D'Angelo, G.B.Rossi	Endoscopy, NCI
R.V. Iaffaioli	Abdominal Medical Oncology, NCI
S. Lastoria, L Aloj	Nuclear Medicine, NCI
M.C. Piccirillo	Clinical Trial Unit, NCI
E. Di Gennaro	Experimental Pharmacology, NCI
N. Maurea	Cardiology, NCI
F.Tatangelo, G.Botti	Pathology, NCI
E. Cavalcanti	Clinical Pathology, NCI
P. Maiolino	Pharmacy, NCI
A. Petrillo, C.Granata	Radiology, NCI
C.Gallo	Medical Statistics, Second University of Napoli
To be determined	Representatives of other partecipating Institutions

Coordinating Center: Clinical Trials Unit
National Cancer Institute, Napoli

Coordinating Independent Ethical Committee: National Cancer Institute, Napoli

AMENDMENTS

N	Type	Description
1	Substatial	Update of allowed and not allowed concomitant medications

Table of contents

Acronyms 5
Synopsis 7
1. Rationale of the study 11
   1.1. Colorectal cancer treatment options 11
   1.2. Rationale for proposing Regorafenib as second-line treatment 11
   1.3. Rationale for the biologic pharmacogenetic and pharmacokinetic study 12
2. AIMS OF THE STUDY 14
   2.1. Primary aim 14
   2.2. Secondary aims 14
3. SELECTION OF PATIENTS 15
   3.1. Study population 15
   3.2. Inclusion criteria 15
   3.3. Exclusion criteria 15
4. STUDY DESIGN 17
5. TREATMENT PLAN 18
   5.1. Regorafenib treatment 18
   5.2 Dose modifications and interruptions 18
   5.3. Definitive interruption of study treatment 26
   5.4. Drug logistics and accountability 26
   5.5. Concomitant treatment 27
6. STUDY PROCEDURES 30
   6.1 Screening visits/procedures 30
   6.2 Treatment visits/procedures 31
   6.3 End-of-treatment visit 32
   6.3. Follow-up 33
   6.4. Collection of CT scans 33
7. RESPONSE EVALUATION 37
   7.1. Measurability of tumour lesions 37
   7.2. Identification of "target" and "non-target" lesions 37
   7.3. Evaluation of target lesion response 38
   7.4. Evaluation of non target lesion response 38
   7.5. Evaluation of best overall response 39
   7.6. Reporting of results 39
8. ADVERSE EVENTS 40
   8.1. Definitions 40

8.2. Definition of a serious adverse event 40
8.3. Collection and reporting of adverse events 41
8.3. Intensity of adverse events 43
8.4. Collection and reporting of serious adverse events (SAE) 43
8.4.4. Outcome 45
8.5. Assessments and documentation of adverse events 45
8.6. Expected adverse events 45
8.7. Procedures for safety reporting 45
8.8. Pregnancies 46
9. STATISTICAL ANALYSIS 47
9.1 Primary endpoint 47
9.2. Secondary endpoints 47
9.3. 1st stage analysis 47
10. BIOMARKERS 48
10.1. Blood sample collection and analysis 48
10.2. Statistical analysis of biomarkers 49
11. TUMOR METABOLIC EVALUATION 50
12. QUALITY ASSURANCE AND MONITORING 51
13. DATA COLLECTION PROCEDURES 51
13.1. Contacts 51
14. PERSONAL DATA PROTECTION PROCEDURES 52
15. ETHICAL CONSIDERATIONS 52
15.1. Risk of undertreatment 52
15.2. Risk of toxicity (overtreatment) 52
16. SUBJECTS INFORMATION AND CONSENT 53
17. ADMINISTRATIVE ASPECTS 54
18. REFERENCES 55
19. APPENDICES 59
19.1. Eastern Cooperative Oncology Group performance status 59
19.2. Glomerular filtration rate 60
19.3. CYP3A4 inhibitors/inducers 61
19.4. Common Terminology Criteria for Adverse Events 62
19.5. New York Heart Association functional classification 63
19.6. Examples of a low-fat breakfast 64

Acronyms

AE: adverse event
AIFA: Agenzia Italiana del Farmaco
Ang-2: angiopoietin-2
BP: blood pressure
CBC: complete bood count
CEC: circulating endothelial cell
CEP: circulating endothelial precursor cell
cfDNA: circulating free DNA
CR: complete response
CRC: colorectal cancer
eCRF: electronic case report form
CRM: circumferential resection margin
CT: computer tomography
CTCAE: Common Terminology Criteria for Adverse Events
CYP: cytochrome P
D: day
DPD: Dihydropyrimidine deydrogenase
ECOG: Eastern Cooperative Oncology Group
EDTA: Ethylenediaminetetraacetic acid
FDA: Food and Drug Administration
FDG-PET-CT: fluorodeoxyglucose positron emission tomography-computed tomography
G-CSF: granulocyte-colony stimulating factor
GFR: glomerular filtration rate
HDAC: histone deacetylase
HFSR: hand-foot syndrome reaction
IB: investigator brochure
ICF: informed consent form
LVEF: left ventricular ejection fraction
miRNA: microRNA
MRI: magnetic resonance imaging
MTD: maximum tolerated dose
NCI: National Cancer Institute
P: progression
PBMC: peripheral blood mononuclear cell
PD: progressive disease
PDGF: platelet-derived growth factor
PIGF: placental growth factor
PPE: palmar-plantar erythrodysesthesia
PR: partial response
PS: performance status
QoL: quality of life
RECIST: Response Evaluation Criteria In Solid Tumours
ROI: region of interest
SAE: serious adverse event
SD: stable disease

SNP: single nucleotide polymorphism
sTIE: secreted angiopoietins receptor
SUSAR: suspected unexpected serious adverse reaction
SUV: Standardized Uptake Value
TLG: Total Lesion Glycolysis
TP: thymidine phosphorylase
TS: thymidylate synthase
UGT: uridina diphosphate glucuronosyl transferase
ULN: upper limit of normality
US: United States
VEGF: vascular endothelial growth factor
VEGF-R: vascular endothelial growth factor receptor
sVEGF-R: secreted vascular endothelial growth factor receptor
sVEGF-R2: secreted vascular endothelial growth factor receptor type 2

Synopsis

OBJECTIVES OF THE STUDY

Primary aim

To assess if regorafenib is active enough, in terms of 6-month progression-free rate, to warrant further comparative studies in patients with RAS-mutant advanced colorectal cancer who have progressed after first-line oxaliplatin-based chemotherapy plus bevacizumab.

Secondary aims

To describe:

- toxicity
- objective response rate
- progression free-survival
- overall survival.

To explore the prognostic and predictive value of:

- circulating endothelial (CEC) and progenitor (CEP) cells (at baseline and during treatment)
- circulating angiogenic factors (i.e. angiopoietin, sTIE, Ang-2, VEGF isoforms, PDGF, PIGF, sVEGF-R-1 and -2 isoforms)
- circulating cytokines profiling
- circulating miRNA profiling
- circulating free DNA (cfDNA) mutations
- VEGF, VEGFR, CYP3A4 and UTG1A9 polymorphisms
- early assessment of metabolic response with PET-CT scan.

SELECTION OF PATIENTS

Inclusion criteria

1. Histologically confirmed diagnosis of colorectal adenocarcinoma
2. Any RAS mutation that prevent treatment with anti-EGFR antibodies
3. Stage IV
4. Measurable disease according to RECIST v. 1.1
5. Disease progression during or following a treatment with fluoropyrimidine, oxaliplatin and bevacizumab, and a treatment with irinotecan is not considered immediately mandatory by the Investigator
6. Age ≥ 18 years
7. ECOG Performance Status 0-1
8. Neutrophils $\geq$ 1,5 x 10^9/L, platelets $\geq$ 100 x 10^9/L, and hemoglobin $\geq$ 9 g/dL without transfusion or granulocyte-colony stimulating factor (G-CSF) and other hematopietic growth factors.
9. Bilirubin level $\leq$ 1.5 x ULN
10. Glomerular filtration rate $\geq$ 30 mL/min/1.73 m^2 according to the Modified Diet in Renal Disease abbreviated formula
11. AST (SGOT) and ALT (SGPT) ≤ 3.0 x ULN (≤ 5 x ULN if liver metastasis are present)

12. Alkaline phosphatase ≤ 2.5 x ULN (≤ 5 x ULN if liver metastasis are present)
13. Serum creatinine ≤ 1.5 x ULN
14. Amilase and lipase ≤ 1.5 x ULN
15. INR and aPTT ≤ 1.5 x ULN. Subjects who are therapeutically treated with an agent such as warfarin or heparin will be allowed to participate if no underlying abnormality in coagulation parameters exists per medical history.
16. Understand, be willing to give consent, and sign the written informed consent form (ICF) prior to undergoing any study-specific procedure.
17. If female and of childbearing potential, have a negative result on a pregnancy test performed a maximum of 7 days before initiation of study treatment.
18. If female and of childbearing potential, or if male, agree to use adequate contraception (eg, abstinence, intrauterine device, oral contraceptive, or double-barrier method) based on the judgment of the investigator or a designated associate from the date on which the ICF is signed until 8 weeks after the last dose of study drug.
19. Life expectancy of greater than 3 months

Exclusion criteria

1. Previous treatment with regorafenib or irinotecan
2. Are taking strong cytochrome P (CYP) CYP3A4 inhibitors (eg, clarithromycin, indinavir, itraconazole, ketoconazole, nefazodone, nelfinavir, posaconazole, ritonavir, saquinavir, telithromycin, voriconazole) or strong CYP3A4 inducers (eg, carbamazepine, phenobarbital, phenytoin, rifampin, St. John’s Wort)
3. Have had a major surgical procedure, open biopsy, or significant traumatic injury within 28 days prior to initiation of study treatment
4. Have congestive heart failure classified as New York Heart Association Class 2 or higher
5. Have had unstable angina (angina symptoms at rest) or new-onset angina ≤ 3 months prior to screening.
6. Have had a myocardial infarction < 6 months prior to initiation of study treatment.
7. Have cardiac arrhythmias requiring anti-arrhythmic therapy, with the exception of beta blockers or digoxin.
8. Have had arterial or venous thrombotic or embolic events such as cerebrovascular accident (including transient ischemic attacks), deep vein thrombosis, or pulmonary embolism within 6 months prior to the initiation of study treatment
9. Symptomatic brain metastases or meningeal tumors
10. Patients with evidence or history of bleeding diathesis
11. Uncontrolled hypertension (systolic blood pressure [SBP] >140 mmHg or diastolic blood pressure [DBP] > 90 mmHg)
12. Have interstitial lung disease with ongoing signs and symptoms at the time informed consent is obtained

13. Have persistent proteinuria > 3.5 g/24 hours measured by urine protein creatinine ratio from a random urine sample (≥ Grade 3, NCI-CTCAE v 4.0).
14. Have unresolved toxicity higher than National Cancer Institute-Common Terminology for Adverse Events version 4.0 (NCI-CTCAE v 4.0) Grade 1 attributed to any prior therapy/procedure, excluding alopecia and/or oxaliplatin-induced neurotoxicity ≤ Grade 2 and hemoglobin ≥ 9 g/dL as per inclusion criteria
15. Patients who cannot take oral medication, who require intravenous alimentation, have had prior surgical procedures affecting absorption, or have active peptic ulcer disease
16. Pregnant or lactating women
17. Any other malignancies within 5 years (except for adequately treated carcinoma in situ of the cervix or non melanoma skin cancer)
18. Any unstable systemic disease (including active infections, any significant hepatic, renal or metabolic disease), metabolic dysfunction, physical examination finding, or clinical laboratory finding that contraindicates the use of regorafenib or render the patient at high risk for treatment complications
19. Sexually active males and females (of childbearing potential) unwilling to practice contraception during the study.
20. Have any other serious or unstable illness, or medical, psychological, or social condition, that could jeopardize the safety of the subject and/or his/her compliance with study procedures, or may interfere with the subject's participation in the study or evaluation of the study results.
21. Have a known hypersensitivity to any of the study drugs, study drug classes, or excipients in the formulation of the study drugs.
22. Have a close affiliation with the investigational site (eg, be a close relative of the investigator) or be a dependent person (eg, be an employee or student working at the investigational site).

STUDY DESIGN

This is a multicentre, single-arm, phase 2 study according to Simon's two-stage optimal design. This design has been applied to minimize the number of patients exposed to a possibly inactive drug, considering that alternative treatment options exist, although not satisfactory and likely to be more toxic.
The primary endpoint will be the rate of patients alive and not progressed at 6 months.
The sample size calculation is based on the following statistical parameters:

- minimum acceptable rate of patients alive without progression at 6 months (p0) = 30%;
- auspicated rate (p1) = 50%
- type I and II errors = 10%.

According to these parameters, 22 evaluable patients should be enrolled at the first stage and the study will proceed to the second stage if at least 8 patients will be alive without progression after 6 months. At the second stage, further 24 evaluable patients will be enrolled for an overall sample of 46 evaluable patients. The study will be considered positive if at least 18 patients out of 46 will be alive without progression at 6 months. It is planned that 5 to 10 centres will participate in the study, which could be completed in 24 months

EXPERIMENTAL TREATMENT PLAN

Regorafenib

Patients will receive regorafenib orally 160 mg once daily for the first 3 weeks of each 4-week cycle.
Treatement will be definitively stopped in case of disease progression, unacceptable toxic effects, motivated decision to stop the treatment by the treating physician, or refusal or withdrawal of consent by the patient.

RESPONSE EVALUATION

Response will be codified according to the RECIST (Response Evaluation Criteria In Solid Tumours) guidelines version 1.1.

SAFETY EVALUATION

All adverse events have to be reported in the toxicity case report form, graded according to the Common Terminology Criteria for Adverse Events of the National Cancer Institute (CTCAE-NCI) version 4.0.

ADMINISTRATIVE ASPECTS

The study is a non-profit investigator initiated trial.
The National Cancer Institute of Napoli is the non-profit sponsor of the study and will be responsible for protocol development, regulatory approvals, data collection, drug distribution and pharmacovigilance, monitoring, data analysis and publication.
The experimental drug will be provided for free by the manufacturer.
The sponsor will provide an insurance policy to cover possible damages caused to patients participating in the trial.
The study is planned to be multicentre in Italy and 5 to 10 centres are expected to participate.

1. RATIONALE OF THE STUDY

1.1. Colorectal cancer treatment options

Colorectal cancer (CRC) is one of the most commonly diagnosed malignancies worldwide, comprising approximately 13% of all new cancer diagnoses in Europe, and it is the second leading cause of cancer deaths in both genders (1). Around one quarter of patients with CRC present with metastatic disease at time of diagnosis and a further third of patients will develop metastases during the course of their disease. The treatment of patients with metastatic CRC has considerably improved since the 1990s when 5-Fluorouracil was the only effective treatment option available, second line therapy did not essentially exist and the median survival of metastatic patients was approximately 12 months (2). The introduction into clinical practice of new cytotoxics (irinotecan and oxaliplatin) and molecular target agents (bevacizumab, cetuximab and panitumumab) has generated new treatment options (3) (4) (5) (6) (7). As a result, median overall survival of patients with metastatic CRC is doubled and up to 70% of patients can receive second line treatment (8) (9). Standard treatment for metastatic CRC commonly includes a fluoropyrimidine-based chemotherapy regimen with oxaliplatin or irinotecan and a monoclonal antibody targeting vascular endothelial growth factor (VEGF), bevacizumab, or epidermal growth factor receptor (EGFR), cetuximab and panitumumab, with a switch to the alternate chemotherapy regimen and monoclonal antibody in second line. However, treatment options are limited in patients whose tumor has *RAS* mutations, since these patients do not have any benefit with anti-EGFR treatments (10) (11) (12). Currently, patients with *RAS* mutant disease treated with bevacizumab and oxaliplatin-based first-line chemotherapy can receive at progression only the alternative irinotecan-based chemotherapy. However, the recent VELOUR and ML18147 trials, which introduced the new biological concept that maintaining angiogenesis inhibition is beneficial for patients with metastatic CRC (13) (14), provide new second-line treatment options for these patients. Indeed, these trials show respectively the benefit of the new VEGF-targeted agent aflibercept in combination with Folfiri regimen in second-line setting independently of prior use of bevacizumab, and the efficacy of continuing bevacizumab, switching only chemotherapy, after failure of a bevacizumab-based first-line treatment, independently of *KRAS* status (15).

1.2. Rationale for proposing Regorafenib as second-line treatment

Regorafenib is an oral multikinase inhibitor that blocks the activity of multiple protein kinases, including kinases involved in the regulation of tumor angiogenesis (i.e. VEGFR1, VEGFR2, VEGFR3 and TIE2) and oncogensis (i.e. KIT, RET, RAF1, BRAF and BRAFV600E) and tumor microenvironment (PDGFR and FGFR). Regorafenib has shown antitumor activity in colorectal cancer models in preclinical studies (16). A recent phase III trial, the CORRECT study, demonstrated a benefit in survival for patients treated with regorafenib versus those receiving only best supportive care, after progression to all available treatments (17). Interestingly, survival benefit was independent of

KRAS status. Moreover, regorafenib showed an acceptable safety profile, with adverse events easily manageable with dose reduction or interruption, and no negative effect on quality of life. These results provide further evidence for maintaining angiogenesis inhibition after disease progression and offer regorafenib as a new line of therapy in this refractory population.
On these bases, also considering the advantages of an oral drug, we aim to explore the value of regorafenib in more early-stage metastatic CRC.
We plan a single-arm phase II study of regorafenib monotherapy to test activity and safety, in second-line treatment of patients with *KRAS* mutant metastatic CRC previously treated with oxaliplatin-based chemotherapy plus bevacizumab.
The study will include several correlative studies since there are unmet needs for a better elucidation of the mechanism of action of regorafenib and for the identification of predictive biomarkers of clinical benefit of regorafenib.

1.3. Rationale for the biologic pharmacogenetic and pharmacokinetic study

Antiangiogenic therapy is currently in clinical trial for treatment of colorectal cancer and other cancers but validate predictive or prognostic biomarkers were not identified yet.
Regorafenib is a multikinase inhibitor that blocks the activity of multiple protein kinases not only kinases involved in the regulation of tumor angiogenesis (i.e. VEGFR1, VEGFR2, VEGFR3 and TIE2) but also in oncogensis such as KIT, RET, RAF1, BRAF and BRAFV600E and tumor microenvironment (PDGFR and FGFR).
Several reports demonstrated that circulating endothelial cells (CEC) count in the peripheral blood (PB) of cancer patients correlates with prognosis and represents a promising tool for selecting patients who might benefit from anti-angiogenic therapies *(18) (19) (20) (21) (22) (23)*. In parallel, chemotherapy mobilization of bone marrow–derived CEP appeared as a consistent finding and a key mechanism mediating tumor resistance (*(19) (24)*). Recent clinical trials suggested that the anti-VEGF antibody (Bevacizumab) was able to decrease CEC and CEP count in patient's PB (25) (26).
Based on our previous experience and our preliminary data on the evaluation of CECs in cancer patients we hypothesize that CEC counts could represent an important prognostic and predictive biomarker for anti anti-angiogenic drug including Regorafenib.
It has been recently demonstrated that single nucleotide polymorphisms (SNP) in VEGF gene may alter VEGF protein concentrations influencing the process of angiogenesis. Moreover, it had been also demonstrated that VEGF SNPs plays an important role in the risk of recurrence, prognosis and survival of colorectal cancer *(27)* as well as in the response to bevacizumab treatment. On these bases we will also perform VEGF SNPs analysis on DNA collected from patient blood- derived cells.
Regorafenib is metabolised by CYP3A4 and by uridina diphosphate glucuronosyl transferase (UGT1A9). It had been recently identified a new SNP in intron 6 of CYP3A4 gene, with a frequency of 5-7% in the Caucasian population, associated with low hepatic CYP3A4 expression and activity (28).

Despite, genotyping for some pharmacogenetic polymorphisms or mutations implicated in drug metabolism or resistances are identified, their analysis prior to drugs prescription remains uncommon. Therefore, the identification of SNPs of CYP3A4 and/or UGT1A9 in patients undergoing regorafenib treatment could improve novel mechanism of resistance or response to this antiangiogenic drug. Multiplex technologies offer a noninvasive, easy and convenient method of simultaneously assessing a much larger number of biologically relevant cytokine and angiogenic factor from small plasma volumes. Moreover, the high stability of DNA and miRNA in the plasma of patients with cancer and their correlation with the expression in the tumor, suggest the possibility to identify innovative predictive biomarkers of benefit or resistence for anti-angiogenic therapy.
All together the data obtained with different types of circulating biomarkers together with imaging approach such as FDG-PET, could help to select the patients most likely to benefit from regorafenib therapy, and to identify possible mechanisms of resistance and consequently new targets to define innovative therapeutic strategy for improving anti-angiogenic therapy.

2. AIMS OF THE STUDY

2.1. Primary aim

To assess if regorafenib is active enough, in terms of 6-month progression-free rate, to warrant further comparative studies in patients with RAS-mutant advanced colorectal cancer who have progressed after first-line oxaliplatin-based chemotherapy plus bevacizumab.

2.2. Secondary aims

To describe:

- toxicity
- objective response rate
- progression free-survival
- overall survival.

To explore the prognostic and predictive value of:

- circulating endothelial (CEC) and progenitor (CEP) cells (at baseline and during treatment)
- circulating angiogenic factors (i.e. angiopoietin, sTIE, Ang-2, VEGF isoforms, PDGF, PIGF, sVEGF-R-1 and -2 isoforms)
- circulating cytokines profiling
- circulating miRNA profiling
- circulating free DNA (cfDNA) mutations
- VEGF, VEGFR, CYP3A4 and UTG1A9 polymorphisms
- early assessment of metabolic response with PET-CT scan.

3. SELECTION OF PATIENTS

3.1. Study population

Patients with RAS mutant advanced colorectal cancer who have progressed after first-line oxaliplatin-based chemotherapy plus bevacizumab, and for whom an irinotecan-based treatment is not considered immediately mandatory by the Investigator.

3.2. Inclusion criteria

1. Histologically confirmed diagnosis of colorectal adenocarcinoma
2. Any RAS mutation that prevent treatment with anti-EGFR antibodies
3. Stage IV
4. Measurable disease according to RECIST v. 1.1
5. Disease progression during or following a treatment with fluoropyrimidine, oxaliplatin and bevacizumab, and a treatment with irinotecan is not considered immediately mandatory by the Investigator
6. Age ≥ 18 years
7. ECOG Performance Status 0-1
8. Neutrophils ≥ 1,5 x 109/L, platelets ≥ 100 x 109/L, and hemoglobin ≥ 9 g/dL without transfusion or granulocyte-colony stimulating factor (G-CSF) and other hematopietic growth factors.
9. Bilirubin level ≤ 1.5 x ULN
10. Glomerular filtration rate ≥ 30 mL/min/1.73 m2 according to the Modified Diet in Renal Disease abbreviated formula
11. AST (SGOT) and ALT (SGPT) ≤ 3.0 x ULN (≤ 5 x ULN if liver metastasis are present)
12. Alkaline phosphatase ≤ 2.5 x ULN (≤ 5 x ULN if liver metastasis are present)
13. Serum creatinine ≤ 1.5 x ULN
14. Amilase and lipase ≤ 1.5 x ULN
15. INR and aPTT ≤ 1.5 x ULN. Subjects who are therapeutically treated with an agent such as warfarin or heparin will be allowed to participate if no underlying abnormality in coagulation parameters exists per medical history.
16. Understand, be willing to give consent, and sign the written informed consent form (ICF) prior to undergoing any study-specific procedure.
17. If female and of childbearing potential, have a negative result on a pregnancy test performed a maximum of 7 days before initiation of study treatment.
18. If potentially childbearing female, or if male, agree to use adequate contraception (eg, abstinence, intrauterine device, oral contraceptive, or double-barrier method) from the date on which the ICF is signed until 8 weeks after the last dose of study drug.
19. Life expectancy of greater than 3 months

3.3. Exclusion criteria

1. Previous treatment with regorafenib or irinotecan
2. Are taking strong cytochrome P (CYP) CYP3A4 inhibitors (eg, clarithromycin, indinavir, itraconazole, ketoconazole, nefazodone,

nelfinavir, posaconazole, ritonavir, saquinavir, telithromycin, voriconazole) or strong CYP3A4 inducers (eg, carbamazepine, phenobarbital, phenytoin, rifampin, St. John's Wort)

3. Have had a major surgical procedure, open biopsy, or significant traumatic injury within 28 days prior to initiation of study treatment
4. Have congestive heart failure classified as New York Heart Association Class 2 or higher
5. Have had unstable angina (angina symptoms at rest) or new-onset angina ≤ 3 months prior to screening.
6. Have had a myocardial infarction < 6 months prior to initiation of study treatment.
7. Have cardiac arrhythmias requiring anti-arrhythmic therapy, with the exception of beta blockers or digoxin.
8. Have had arterial or venous thrombotic or embolic events such as cerebrovascular accident (including transient ischemic attacks), deep vein thrombosis, or pulmonary embolism within 6 months prior to the initiation of study treatment
9. Symptomatic brain metastases or meningeal tumors
10. Patients with evidence or history of bleeding diathesis
11. Uncontrolled hypertension (systolic blood pressure [SBP] >140 mmHg or diastolic blood pressure [DBP] > 90 mmHg)
12. Have interstitial lung disease with ongoing signs and symptoms at the time informed consent is obtained
13. Have persistent proteinuria > 3.5 g/24 hours measured by urine protein creatinine ratio from a random urine sample (< Grade 3, CTCAE v 4.0).
14. Have unresolved toxicity higher than National Cancer Institute-Common Terminology for Adverse Events version 4.0 (CTCAE v 4.0) Grade 1 attributed to any prior therapy/procedure, excluding alopecia and/or oxaliplatin-induced neurotoxicity ≤ Grade 2 and hemoglobin ≥ 9 g/dL as per inclusion criteria
15. Patients who cannot take oral medication, who require intravenous alimentation, have had prior surgical procedures affecting absorption, or have active peptic ulcer disease
16. Pregnant or lactating women
17. Any other malignancies within 5 years (except for adequately treated carcinoma in situ of the cervix or non melanoma skin cancer)
18. Any unstable systemic disease (including active infections, any significant hepatic, renal or metabolic disease), metabolic dysfunction, physical examination finding, or clinical laboratory finding that contraindicates the use of regorafenib or render the patient at high risk for treatment complications
19. Sexually active males and females (of childbearing potential) unwilling to practice contraception during the study.
20. Have any other serious or unstable illness, or medical, psychological, or social condition, that could jeopardize the safety of the subject and/or his/her compliance with study procedures, or may interfere with the subject's participation in the study or evaluation of the study results.
21. Have a known hypersensitivity to any of the study drugs, study drug classes, or excipients in the formulation of the study drugs.

22. Have a close affiliation with the investigational site (eg, be a close relative of the investigator) or be a dependent person (eg, be an employee or student working at the investigational site).

4. STUDY DESIGN

This is a multicentre, single-arm, phase 2 study according to Simon's two-stage optimal design. This design has been applied to minimize the number of patients exposed to a possibly inactive drug, considering that alternative treatment options exist, although not satisfactory and likely to be more toxic. Patients who will receive at least one dose of study drug will be considered evaluable.

The primary endpoint will be the rate of evaluable patients alive and not progressed at 6 months.

The sample size calculation is based on the following statistical parameters:

- minimum acceptable rate of patients alive without progression at 6 months (p0) = 30%;
- auspicated rate (p1) = 50%
- type I and II errors = 10%.

According to these parameters, 22 evaluable patients should be enrolled at the first stage and the study will proceed to the second stage if at least 8 patients will be alive without progression after 6 months. At the second stage, further 24 evaluable patients will be enrolled for an overall sample of 46 evaluable patients. The study will be considered positive if at least 18 patients out of 46 will be alive without progression at 6 months. It is planned that 5 to 10 centres will participate in the study, which could be completed in 24 months.

5. TREATMENT PLAN

5.1. Regorafenib treatment

Patients will receive regorafenib orally 160 mg once daily for the first 3 weeks of each 4-week cycle.
Treatment will be definitively stopped in case of disease progression, unacceptable toxic effects, motivated decision to stop the treatment by the treating physician, or refusal or withdrawal of consent by the patient.

5.2 Dose modifications and interruptions

The dose or dosing schedule of the study drug may be modified following the occurrence of a clinically significant AE that is:

- related or not related to the study drug, and
- of a pre-specified severity Grade

All dose modifications are to be made according to the pre-defined dose levels presented in Table 1.

Table1 : Pre-defined dose levels

Dose level	Number of tablets
Dose level 0 (standard dose)	4 regorafenib 40-mg tablets (160 mg)
Dose level -1	3 regorafenib 40-mg tablets (120 mg)
Dose level -2	2 regorafenib 40-mg tablets (80 mg)

Dose reductions leading to a daily dose below Dose Level -2 (ie, fewer than 2 tablets daily) are not permitted. Treatment will be definitively discontinued if the subject is unable to tolerate a daily dose of at least two tablets.

Treatment with the study drug will be also definitively discontinued if it is necessary to interrupt the study drug treatment schedule for more than one cycle (4 consecutive weeks that is maximum of 5 weeks permitted between doses of regorafenib).

5.2.1. Recommended dose modifications

Recommended dose modifications (dose reduction and dose interruption) for general and special situations are provided in Table2, Table3, Table4 and Table5.

5.2.2. Conflicting recommendations for dose modification

A subject may simultaneously experience several clinically significant toxicities requiring dose modification. If the recommendations for dose modification related to the simultaneously experienced toxicities conflict with one another, the investigator should select the dose modification resulting in the lowest dose level.

5.2.3. Dose re-escalation

The maximum dose level is 0 (4 tablets daily). If dose reduction is required to Dose Level -1 (3 tablets daily) or Dose Level -2 (2 tablets daily), the dose may be re-escalated (increased) to Dose Level -1 (3 tablets daily) or Dose Level 0 (4 tablets daily) at the discretion of the investigator, provided the severity of all AEs requiring dose reduction have returned to baseline Grades after a full cycle of therapy. Dose escalation should be done one dose level at a time.

The exception to this rule occurs with toxicities associated with HFSR, also known as PPE. For subjects with HFSR, dose re-escalation may be considered, at the discretion of the investigator, after AE severity is reduced to Grade ≤1. Additional details are provided below (*Dose modifications and supportive measures for HFSR*) and in Table 3.

5.2.4. Dose modifications: general and special recommendations

<u>*General recommendations*</u>

Recommended dose modifications are shown in Table 2 for any clinically significant toxicity that:

- is considered to be related to the study drug,
- has a severity grade ≥ 3 (as defined in NCI-CTCAE v 4.0), and
- is not covered by a special recommendation.
- in addition to the recommended dose modifications, subjects who develop diarrhea, mucositis, anorexia or other events predisposing to fluid loss or inadequate fluid intake should be carefully monitored and rehydrated as clinically necessary to minimize the risk of postural hypotension and renal failure.

<u>*Special recommendations*</u>

- Recommended dose modifications for HFSR (PPE syndrome) with severity Grade ≥ 1 are provided in Table 3.
- Required dose modifications for ALT and/or AST increases related to study drug with severity Grade ≥ 1 (CTCAE v 4.0) are provided in Table 4; recommended liver function monitoring schedules are also provided.
- Recommended dose modifications for treatment-emergent hypertension with severity Grade ≥ 1 (CTCAE v 4.0) are provided in Table 5; general recommendations for the management of hypertension are also provided.

5.2.5. Dose modification/interruption for AEs related to the study drug (except HFSR, hypertension, and liver function test abnormalities)

As shown in Table 2, a study drug-related Grade 3 toxicity (except HFSR, hypertension, and liver function test abnormalities) requires interruption of dosing until the toxicity severity is reduced to ≤ Grade 2. Treatment is then to be restarted at the dose level immediately below the original dose level. If the toxicity severity returns to baseline, dose escalation may be considered at the discretion of the investigator. A second occurrence of an AE with a severity of Grade 3 requires a permanent reduction of dose level. An AE with a severity of Grade 4 requires dose interruption until the severity is reduced to ≤ Grade 2 and a permanent dose reduction of one dose level. All dermatological AEs, except for HFSR, should be managed according to the guidelines in Table 2.

Table 2: Dose modification/interruption for adverse events related to the study drug* (except hand-foot skin reaction, hypertension, and liver function test abnormalities)

Severity Grade (CTCAE v 4.0)	Dose Interruption	Dose Reduction	Dose for Subsequent Cycles
Grade 0-2	Treat on time	No change	No change
Grade 3	Delay until ≤ Grade 2[a]	Reduce by 1 dose level	If toxicity remains ≤ Grade 2, dose re-escalation may be considered at the discretion of the treating investigator. If dose is re-escalated and toxicity (≥ Grade 3) recurs, institute permanent dose reduction
Grade 4	Delay until ≤ Grade 2[a]	Reduce by 1 dose level. Permanent discontinuation may be considered at the investigator's discretion.	---

* Excludes alopecia, non-refractory nausea/vomiting, non-refractory hypersensitivity, and non-clinically significant and asymptomatic laboratory abnormalities

a: If no recovery after one cycle (4 consecutive weeks; maximum of 5 weeks between doses of regorafenib), treatment should be permanently discontinued.

5.2.6. Dose modifications and supportive measures for HFSR

Prior to beginning treatment with study drug, measures that may prevent or minimize the severity of HFSR should be undertaken. If HFSR of severity Grades 1 or 2 occurs during treatment, any preventive or supportive measures not already taken should be instituted immediately. These include the following:

1. <u>Control of calluses</u>

Before initiating treatment with the study drug:

- Check condition of hands and feet.
- Suggest a manicure/pedicure, when indicated.
- Recommend pumice stone use for callus or “rough spot” removal.
- During regorafenib treatment:
- Avoid pressure points.
- Avoid items that rub, pinch, or create friction.

2. <u>Use of creams</u>

- Non-urea-based creams: apply liberally
- Keratolytic creams: use sparingly and apply only to affected (hyperkeratotic) areas.
- Urea-based creams
- Salicylic acid 6%
- Alpha hydroxy acid-based creams
  - Creams containing approximately 5% to 8% alpha hydroxyl acid provide gentle chemical exfoliation.
  - Apply liberally twice daily.
- Topical analgesics, such as lidocaine 2%, should be considered for pain control.
- High-potency topical corticosteroids, such as clobetasol 0.05%, should be considered for subjects with HFSR Grade 2 or 3. Avoid systemic steroids.

3. <u>Cushions</u>

Protect tender areas:

- Use socks/gloves to cover moisturizing creams.
- Wear well-padded footwear.
- Use insole cushions or inserts (eg, silicon, gel).
- Foot soaks with tepid water and Epsom salts

If supportive measures do not produce the desired effects, dose modification, as outlined in Table 3, is the next step. Dose interruption (7 days) followed by

restarting treatment at Dose Level -1 may be instituted for Grade 2 toxicity if supportive measures do not reduce the severity to ≤ Grade 1 within 7 days.

For subjects with Grade 3 HFSR, at the first occurrence a 1-level dose reduction is recommended; at the second occurrence a decrease of an additional dose level is recommended.

Study treatment is to be discontinued for the fourth occurrence of HFSR Grade 2 and for the third occurrence of HFSR Grade 3.

For subjects who require a dose reduction for Grade 2 or 3 HFSR, the dose of study drug may be increased to the starting dose after one full cycle of therapy has been administered at the reduced dose without the appearance of HFSR > Grade 1. Dose escalation should be done one dose level at a time.

In general, a more liberal management for dose modification is allowed if judged by the investigator to be medically appropriate (eg, dose reduction for Grade 1 HFSR).

Table 3: Dose modification/interruption and toxicity grading for hand-foot skin reaction (palmar-plantar erythrodysesthesia syndrome)

Skin Toxicity Grade (CTCAE v 4.0)	Occurrence	Suggested Dose Modification/Interruption[a]
Grade 1: Numbness, dysesthesia, paraesthesia, tingling, painless swelling, erythema or discomfort of the hands or feet that does not disrupt the subject's normal activities	Any	Maintain dose level and institute supportive measures immediately for symptomatic relief.
Grade 2: Painful erythema and swelling of the hands or feet and/or discomfort that affects the subject's normal activities	1st occurrence	Institute supportive measures immediately. Consider decreasing dose by one dose level; if no improvement, interrupt therapy for 7 days or until toxicity ≤ Grade 1.
	No improvement at 7 days or 2nd occurrence	Interrupt therapy ≤ 28 days[a] or until toxicity ≤ Grade 1. When resuming treatment, reduce dose by one dose level.
	3rd occurrence	Interrupt therapy ≤ 28 days[a] or until toxicity ≤ Grade 1. When resuming treatment, reduce dose by one additional dose level.[b,c]
	4th occurrence	Discontinue treatment

Table 3: Dose modification/interruption and toxicity grading for hand-foot skin reaction (palmar-plantar erythrodysesthesia syndrome)

Skin Toxicity Grade (CTCAE v 4.0)	Occurrence	Suggested Dose Modification/Interruption[a]
Grade 3: Moist desquamation, ulceration, blistering or severe pain of the hands or feet, or severe discomfort that causes the subject to be unable to work or perform activities of daily living	1st occurrence	Institute supportive measures immediately. Interrupt therapy for 7 days or until toxicity ≤ Grade 1. When resuming treatment, decrease dose by one dose level.[c]
	2nd occurrence	Institute supportive measures immediately. Interrupt therapy for a minimum of 7 days but ≤ 28 days[a] or until toxicity ≤ Grade 1. When resuming treatment, decrease dose by one additional dose level.[b,c]
	3rd occurrence	Discontinue treatment permanently.

In general, a more liberal management for dose modification is allowed if judged by the investigator to be medically appropriate (eg, dose reduction for Grade 1 hand-foot skin reaction).

a. Dose interruption for ≥ 28 days (one cycle [4 consecutive weeks; maximum of 5 weeks between doses of regorafenib]) requires permanent discontinuation of study drug.
b. The lowest recommended dose is 80 mg.
c. If toxicity ≤ Grade 1 after dose reduction for one full cycle of therapy, dose re-escalation is permitted at the investigator's discretion.

5.2.7. Dose modification for increases in ALT and/or AST

Dose modifications required for study drug-related increases in ALT and/or AST concentrations and requirements for monitoring are provided in Table 4. During the first 2 cycles of treatment, ALT, AST, and bilirubin must be obtained at baseline and monitored weekly (ie, on Days 1, 8, 15, and 22 of Cycles 1 and 2 [±3 days]), even if values are normal. The investigators may use investigational site or local laboratory AST, ALT, and bilirubin values for subject treatment decisions for Days 8 and 22 of Cycles 1 and 2; these values must be entered in the eCRF

Table 4: Dose modification/interruption for alanine aminotransferase and/or aspartate aminotransferase increases related to study drug

Increases in AST/ALT (CTCAE v 4.0)	1st occurrence	Restart	Recurrence
AST and/or ALT ≤ 5 X ULN (<Grade 3)	Continue dosing, with weekly monitoring of liver function until transaminases return to < 3 X ULN (≤Grade 1) or baseline.		
ALT and/or AST > 5 X ULN (≥Grade 3)	Interrupt dosing, with weekly monitoring until transaminases return to < 3 X ULN or baseline.	If the potential benefit for reinitiating regorafenib is considered to outweigh the risk of hepatotoxicity: reduce 1 dose level and measure serum transaminases weekly for at least 4 weeks.	Discontinue.
ALT and/or AST > 20 X ULN (≥Grade 4)	Discontinue.		
ALT and/or AST > 3 X ULN (≥Grade 2) with concurrent bilirubin > 2 X ULN	Discontinue treatment and measure serum transaminases weekly until resolution. Exception: subjects with Gilbert's syndrome who develop elevated transaminases should be managed as per the recommendations outlined above for ALT/AST elevations.		

ALT, AST, and bilirubin must be obtained at baseline and monitored weekly for the first 2 cycles (ie, on Days 1, 8, 15, and 22 of Cycles 1 and 2 [±3 days]), even if values are normal. The investigators may use investigational site or local laboratory AST, ALT, and bilirubin values for subject treatment decisions for Days 8 and 22 of Cycles 1 and 2; these values must be entered in the eCRF.

5.2.8. Dose modification/interruption and management of treatment-emergent hypertension

Blood pressure (BP) should be monitored on every visit. The recommendations for dose modification/interruption and management of treatment-emergent hypertension are summarized in Table5. Management includes a stepped-care approach to the control of BP as well as dose modification of the study drug if required. The selection of antihypertensive medication used in this setting will be made at the investigator's discretion, considering possible site-specific guidelines. All antihypertensive medications must be entered in the subject's Concomitant Medication eCRF.

Table 5: Dose modification/interruption and management of treatment-emergent hypertension

Severity Grade (CTCAE v4.0)	Definition	Antihypertensive therapy	Dose modification/ interruption
Grade 1	Prehypertension:<br>SBP 120-139 mmHg or DBP 80-89 mmHg	None	Continue study drug.<br>Consider increased BP monitoring.
Grade 2	SBP 140-159 mmHg or DBP 90-99 mmHg<br>**OR**<br>Symptomatic increase in DBP >20 mmHg or to >140/90 mmHg if previously within normal limits	Treat with the aim to achieve DBP ≤ 90 mmHg.<br>If S/DBP previously within normal limits, start antihypertensive monotherapy.<br>If subject is taking antihypertensive medication, titrate the dosage up.	Continue study drug.<br>If symptomatic, delay dose until symptoms resolve AND diastolic BP ≤ 90 mmHg.[a]<br>Restart dose at same dose level.
Grade 3	SBP ≥ 160 mmHg or DBP ≥ 100 mmHg<br>**OR**<br>≥1 antihypertensive drug or more intensive antihypertensive therapy required than previously	Treat to DBP ≤ 90 mmHg.<br>Start antihypertensive medication.<br>**AND/OR**<br>Increase dose of current antihypertensive medication.<br>**AND/OR**<br>Add additional antihypertensive medication(s).	Delay dose until DBP ≤ 90 mmHg and, if symptomatic, until symptoms resolve.[a]<br>Restart dose at same dose level.<br>If BP not controlled with more intensive therapy, reduce dose to Dose Level -1.[b]<br>If Grade 3 hypertension recurs after dose reduction and antihypertensive therapy, reduce dose to Dose Level -2.[c]
Grade 4	Life-threatening consequences (malignant hypertension): transient or permanent neurologic deficit, hypertensive crisis	———	Discontinue therapy.

In cases in which only one value is >Grade 1 (eg, 155/88 mmHg), the guidelines provided for the highest grade should be followed.

BP = blood pressure, DBP = diastolic blood pressure, SBP = systolic blood pressure.

a. Subjects requiring a delay of study treatment > one cycle (4 consecutive weeks; maximum of 5 weeks between doses of regorafenib) must discontinue study drug.
b. If BP remains controlled for ≥ one full cycle, dose re-escalation is permitted at the investigator's discretion.
c. Subjects requiring dose reduction below Dose Level -2 (80mg) must discontinue study drug.

5.3. Definitive interruption of study treatment

A subject can definitively interrupt study treatment for the following reasons:

- Unequivocal progression of disease as demonstrated on a CT/MRI scan. In the case of equivocal lesions, subjects should continue to receive study drug until progression is confirmed (CT/MRI scan, follow-up).
- If, in the investigator's opinion, continuation in the study would be harmful to the subject's well-being
- At his/her own request or at the request of his/her legally acceptable representative. At any time during study treatment and without giving a reason, a subject or his/her legally acceptable representative may decline further study treatment. The subject will not suffer any disadvantage as a result. The subject will continue to be followed up for safety and survival unless he/she withdraws consent.
- Study drug dose reduction by more than two dose levels
- Study drug dose interruption for more than one cycle (4 consecutive weeks; maximum of 5 weeks between doses of regorafenib)
- Severe allergic reactions, such as exfoliate erythroderma, anaphylaxis, or vascular collapse
- Any other adverse reaction deemed sufficiently serious to warrant discontinuation of treatment by the investigator
- If female, a pregnancy test result consistent with pregnancy
- Use of illicit drugs or other substances that may, in the opinion of the investigator, have a reasonable chance of contributing to toxicity or otherwise confounding the results of the study
- Development of any intercurrent illness or situation that may, in the judgment of the investigator affect assessments of clinical status and study endpoints to a relevant degree
- Development of a second malignancy except for cervical cancer in situ, nonmelanoma skin cancer, or superficial bladder tumors classified as noninvasive tumor (Ta), carcinoma in situ (Tis), or tumor invades lamina propria (T1).
- Any subject who definitively interrupt study treatment will be evaluated at the End-of-treatment Visit conducted 30 days (±7 days) after permanently stopping study treatment.
- In all cases, the reason for definitive interruption of study treatment must be entered in the electronic Case Report Form (eCRF) and in the subject's medical records.

5.4. Drug logistics and accountability

Regorafenib will be stored at the investigational sites according to current laws. The coordinating center will manage drug accountability (including distribution, reception, return and distruction) according to the Standard Operating Procedures of the Clinical trials Unit at the National Cancer Institute of Napoli. All the data on drug accountability will be made available for audit or inspection by the drug manufacturer.

The investigators responsibility is to control the distribution to the patients and the use of the study drug. Study drug accountability must be performed for each cycle. Bottles must be returned to the investigator with all unused medication. Throughout the study, all unused study medication will be accounted for. The information on the number of tablets returned at each cycle will be collected through the electronic CRF. The reason(s) for any dose delay, reduction, or interruption also will be recorded in the CRF.

5.5. Concomitant treatment

5.5.1 Prior and concomitant therapy

All medication which is considered necessary for the subject's welfare, and which is not expected to interfere with the evaluation of the study drug, may be given at the discretion of the Investigator. All medications used during the study must be recorded in the subject's source documentation and in the CRF (including start/stop dates, dose frequency, route of administration, and indication). Disease-specific anti-neoplastic therapies, including kinase inhibitors, chemotherapy, radiation therapy, or surgical intervention, are not allowed during study treatment.

5.5.2. Inhibitors / inducers of CYP3A4

In vitro data indicate that regorafenib is metabolized by cytochrome CYP3A4 and uridine diphosphate glucuronosyl transferase UGT1A9.

Administration of ketoconazole (400 mg for 18 days), a strong CYP3A4 inhibitor, with a single dose of regorafenib (160 mg on Day 5) resulted in an increase in mean exposure (area under the curve, AUC) of regorafenib of approximately 33%, and a decrease in mean exposure of the active metabolites, M-2 (N-oxide) and M-5 (N-oxide and N-desmethyl), of approximately 90%.

Administration of rifampicin (600 mg for 9 days), a strong CYP3A4 inducer, with a single dose of regorafenib (160 mg on Day 7) resulted in a reduction in mean exposure (AUC) of regorafenib of approximately 50%, a 3- to 4-fold increase in mean exposure of the active metabolite M-5, and no change in exposure of active metabolite M-2. Other strong CYP3A4 inducers (eg, phenytoin, carbamazepine, phenobarbital, and St. John's Wort) may also increase metabolism of regorafenib.

Subjects on strong CYP3A4 inhibitors or inducers are not eligible for the study (see Section 5.2). During the study, strong CYP3A4 inhibitors (eg, clarithromycin, indinavir, itraconazole, ketoconazole, nefazodone, nelfinavir, posaconazole, ritonavir, saquinavir, telithromycin, voriconazole) or strong CYP3A4 inducers (eg, carbamazepine, phenobarbital, phenytoin, rifampin, St. John's Wort) are not permitted. A listing of CYP3A4 inhibitors and inducers is presented in Appendix.

5.5.3. UGT1A1 and UGT1A9 substrates

In vitro data indicate that regorafenib, as well as its active metabolites M-2, inhibits glucuronidation mediated by uridine diphosphate glucuronosyl transferases UGT1A1 and UGT1A9, whereas M-5 only inhibits UGT1A1 at concentrations that are achieved in vivo at steady state. Administration of regorafenib with a 5-day break prior to administration of irinotecan resulted in an increase of approximately 44% in mean exposure (AUC) of SN-38, a substrate of UGT1A1 and an active metabolite of irinotecan. An increase in mean exposure (AUC) of irinotecan of approximately 28% was also observed. This indicates that co-administration of regorafenib may increase systemic exposure to UGT1A1 and UGT1A9 substrates.

5.5.4. Breast cancer resistance protein and P-glycoprotein substrates

In vitro data indicate that regorafenib is an inhibitor of breast cancer resistance protein (BCRP) (median inhibition concentration [concentration that reduces the effect by 50%, IC50] values about 40 to 70 nanomolar) and P-glycoprotein (IC50 value of about 2 micromolar).

Co-administration of regorafenib may increase the plasma concentrations of concomitant BCRP substrates, such as methotrexate, fluvastatin, atorvastatin and rosuvastatin. Therefore, it is recommended to monitor patients closely for signs and symptoms of increased exposure to BCRP substrates.

Clinical data indicate that regorafenib has no effect on pharmacokinetics of digoxin, a P-glycoprotein substrate, therefore regorafenib can be given concomitantly with p-glycoprotein substrates, such as digoxin, without a clinically meaningful drug interaction.

5.5.5. CYP isoform-selective substrates

In vitro data indicate that regorafenib is a competitive inhibitor of the cytochromes CYP2C8 (Ki value of 0.6 micromolar), CYP2C9 (Ki value of 4.7 micromolar), and CYP2B6 (Ki value of 5.2 micromolar) at concentrations that are achieved in vivo at steady state (peak plasma concentration of 8.1 micromolar). The in vitro inhibitory potency towards CYP3A4 (Ki value of 11.1 micromolar) and CYP2C19 (Ki value of 16.4 micromolar) was less pronounced.

A clinical probe substrate study was performed to evaluate the effect of 14 days of dosing with 160 mg regorafenib on the pharmacokinetics of probe substrates of CYP2C8 (rosiglitazone) CYP2C9 (S-warfarin), CYP 2C19 (omeprazole) and CYP3A4 (midazolam).

Pharmacokinetic data indicate that regorafenib may be given concomitantly with substrates of CYP2C8, CYP2C9, CYP3A4, and CYP2C19 without a clinically meaningful drug interaction.

5.5.6. Antibiotics

The concentration-time profile indicates that regorafenib and its metabolites may undergo enterohepatic circulation. Co-administration of antibiotics that affect the flora of the gastrointestinal tract may interfere with the enterohepatic circulation of regorafenib and may result in a decreased regorafenib exposure. The clinical significance of these potential interactions is unknown, but may result in a decreased efficacy of regorafenib.

5.5.7. Bile salt-sequestering agents

The concentration-time profile indicates that regorafenib and its metabolites may undergo enterohepatic circulation. These compounds may interact with regorafenib by forming insoluble complexes which may impact absorption (or reabsorption), thus resulting in potentially decreased exposure. The clinical significance of these potential interactions is unknown, but may result in a decreased efficacy of regorafenib.

5.5.8. Permissible concomitant medications and treatments

- Standard therapies for concurrent medical conditions. Prophylactic anti-emetics may be administered according to standard practice.
- Treatment with non-conventional therapies (eg, herbs or acupuncture) or vitamin/mineral supplements is acceptable provided that such agents do not interfere, in the opinion of the investigator, with the study endpoints.
- Bisphosphonates
- Clinical data indicate that regorafenib has no effect on digoxin pharmacokinetics, therefore can be given concomitantly with p-glycoprotein substrates, such as digoxin, without a clinically meaningful drug interaction.
- Subjects taking narrow therapeutic index medications, such as warfarin, quinidine and cyclosporine, should be monitored proactively. A subject treated with warfarin or heparin will be allowed to participate provided that the anticoagulant dose and PT-INR and aPTT values are stable. Close monitoring (evaluation ≥ once weekly) is recommended. If either value is above the therapeutic range, the anti-coagulant dose should be modified and the assessments should be repeated once weekly until the value is stable.
- Contrast agents used in CT or MRI scans (these do not need to be entered in the Concomitant Medications CRF).
- G-CSF and other hematopoietic growth factors may be used during the study for the management of acute toxicity such as febrile neutropenia when clinically indicated or at the discretion of the investigator; however, they may not be substituted for a required dose reduction. Long-term administration of erythropoietin is permitted.

5.5.9. Prohibited concomitant medications and treatments

- Systemic anti-cancer therapy including cytotoxic therapy, signal transduction inhibitors, immunotherapy, and hormonal therapy
- Tyrosine kinase inhibitors
- Bone marrow transplant or stem cell rescue
- Use of biologic response modifiers, such as G-CSF within 3 weeks of study entry
- All traditional medicines with an anti-cancer indication, including traditional Chinese medicine (Appendix 22.3)
- St. John's Wort
- Grapefruit or grapefruit juice

Following dose modification of the study drug, therapeutic monitoring of concomitant medications should be performed as needed in a manner consistent with the local clinical standard of care. In general, subjects should be closely monitored for side effects of all concomitant medications regardless of the path of elimination.

6. STUDY PROCEDURES

6.1 Screening visits/procedures

Screening procedures may be completed during multiple visits but must be completed within the specified timeframes before the first dose of study drug.

Screening procedures to be completed or that must be available <u>within 28 days</u> prior to initiation of study treatment:

- Obtain written ICF. No screening procedures may be performed until written informed consent has been obtained.
- Demographics
- Medical history including allergies and prior conditions
- Histologically confirmed diagnosis of colorectal adenocarcinoma
- Tumor assessment: CT or MRI eligibility scan (chest, abdomen, pelvis, and other suspected sites as applicable) meeting the standard of care for the imaging of the respective organ system(s).
- 12-lead ECG
- Review inclusion/exclusion criteria

Screening procedures to be completed <u>within 14 days</u> prior to initiation of study treatment:

- 18FDG-PET CT scan

Screening procedures to be completed <u>within 7 days</u> prior to initiation of study treatment:

- ECOG performance status
- Physical examination
- Documentation of AEs (CTCAE v 4.0 grading)
- Record all concomitant prescribed and over-the-counter medications (including start/stop dates, dose, and indication). All medications and significant non-drug therapies taken within 14 days prior to the start of study drug should be entered in the eCRF; all concomitant medications taken from this visit until 30 days after the last dose of study drug should be recorded at each study visit.
- Perform the following laboratory evaluations:
    - CBC with differential. Specified platelet count (≥100,000 cells/mm^3), hemoglobin concentration (≥ 9 g/dL), and ANC (≥1500 cells/mm^3) must be met *without* transfusion, or G-CSF and other hematopoietic growth factors
    - Chemistry and electrolyte panels
    - A pregnancy test for women of childbearing potential; a negative result is required before initiating study treatment. More frequent evaluation for pregnancy may be required in certain countries; all local regulations regarding pregnancy testing in clinical study subjects must be followed.
    - A urinalysis by dipstick, and a microscopic analysis if the urine dipstick test shows a change from normal
    - Proteinuria qualification
    - GFR calculation
    - Coagulation panel. Subjects treated with warfarin or heparin will be allowed to participate in the study if no underlying abnormality in coagulation parameters exists per prior history; weekly evaluation of PT-INR/aPTT will be required until stability is achieved (as defined by local standard of care and based on prestudy PT-INR/aPTT values).
- CEC and CEP counts (results will not be communicated to the patient and the treating physician)
- Blood samples to be stored for evaluation of cytokines, circulating angiogenic factors, cfDNA mutations and miRNA plasma levels as well as for pharmacogenetic studies.

6.2 Treatment visits/procedures

See Section Schedule of procedures/assessments.

Each treatment cycle comprises 4 weeks. Regorafenib will be administered on a 3-weeks-on/1-week-off dosing schedule.

During Cycles 1 and 2, study visits will be scheduled on Days 1, 8, 15, and 22 and on Day 1 of each cycle from cycle 3 onwards. From Cycle 3 to the end of treatment, visits on Day 15 may be scheduled at the discretion of the investigator; if scheduled, visits on Day 15 should include the same procedures as were performed on Day 15, Cycle 2 (except for the plasma biomarker sampling).

ALT, AST, and bilirubin must be obtained at baseline and monitored weekly during the first two cycles of treatment with regorafenib (ie, on Days 1, 8, 15, and 22 of Cycles 1 and 2 [±3 days]), even if values are normal. The investigators may use investigational site or local laboratory AST, ALT, or bilirubin values; these values must be entered in the eCRF.

Blood pressure monitoring is required to be completed weekly for the first 6 weeks of treatment.

GFR calculations must be done within 7 days of study start, then on Day 1 for the first 6 cycles, and at EOT. Additional calculations may be done at the discretion of the investigator.

CEC and CEP counts, blood samples to be stored for evaluation of cytokines, circulating angiogenic factors, cfDNA mutations and miRNA plasma levels will be performed on day 14th +/- 2 after starting treatment, before starting the 2nd cycle (day 28) at the first restaging (after 6 weeks) and at progression of the disease

6.3 End-of-treatment visit

The End-of-Treatment assessments should be performed no later than 14 days after study treatment has stopped.

- Determination of disease status: see paragraph 7 (*Response Evaluation)*
- 12-lead ECG
- Documentation of AEs (CTCAE v 4.0 grading)
- Concomitant medications
- ECOG performance status
- Physical examination, including BP measurement
- The following laboratory evaluations will be performed
    - CBC with differential
    - Chemistry and electrolyte panels

  - A coagulation panel, including PT-INR and aPTT
  - GFR calculation
  - AST, ALT, and bilirubin
- Perform drug accountability

6.3. Follow-up

All subjects who finish treatment, whichever the reason, will enter the follow-up. All subjects will be followed until death and data on subsequent treatment will be collected. Follow-up information on tumor status and vital status will be updated every 3 months until death.

6.4. Collection of CT scans

It is required that CT scans or other relevant radiologic examinations used for staging and restaging procedures are collected and made available for eventual revision of response and time-to-progression definition.

Table 6: Schedule of procedures/assessments

Treatment Phase	Screeninga (days relative to enrollment)			Cycle 1 (4 weeks)				Cycle 2 (4 weeks)				Cycle 3[1] →
**Day**	-28 days	-14 days	-7 days	Day 1	Day 8	Day 15	Day 22	Day 1	Day 8	Day 15	Day 22	Day 1
ICF signed [b]	X											
Separate ICF for biologic studies	X											
Allocation of Subject ID No.	X											
Demographics		X										
Medical history		X										
Inclusion/exclusion criteria review		X										
Tumor assessment (CT/MRI scan)		X[c]								X[d]		
12-lead ECG		X								X[f]		X[g]
PET/TC scan		X				X[e]						
AEs and toxicities[h]			X	X---→								X→
Concomitant medications			X	X---→								X→
ECOG performance status			X	X				X				X
Physical examination			X	X[f]				X				X
CBC with differential[i]			X	X[f]		X		X		X		X
Chemistry / electrolyte panel			X	X[f]		X		X		X		X
Blood for CEC and CEP counts[j]			X			X		X				
Blood for biomarkers			X			X		X				
Pregnancy test[k]			X									
Urinalysis by dipstick (with microscopic analysis if indicated)[l]			X	X[f]		X		X		X		X
Proteinuria quantification (if indicated)[l]			X	X[f]		X		X		X		X
GFR calculation[m]			X	X[f]				X				X
Coagulation panel[n]			X	X[f]		X[o]		X		X[n]		X

AST, ALT, bilirubin[p]		X	X	X	X	X	X	X	X	X	
Blood pressure monitoring[q]		X	X	X	X	X	X	X	X		X
Drug dispensing			X				X				X
Drug accountability							X				X

Treatment Phase	End-of-treatment	Safety Follow-up[r]	Survival Follow-up[s]
Days		30 (±7) days after last dose	Q3 months
CT/MRI scan[d]	X		
12-lead ECGg	X		
AEs and toxicities[h]	X	X	
Concomitant medications	X	X	
ECOG performance status	X		
Physical examination	x		
CBC with differential	X	X	
Chemistry / electrolyte panel	X	X	
Blood for CEC and CEP counts[j]	X		
Coagulation panel	X	X	
GFR calculation	X		
AST, ALT and bilirubin	X	X	
Drug accountability	X		
Survival status			X

AE = adverse event, ALT = alanine aminotransferase, AST = aspartate aminotransferase, CEA = carcinoembryonic antigen, CBC = complete blood count, CRC = colorectal cancer, CT = computed tomography, ECG = electrocardiogram, ECOG = Eastern Cooperative Oncology Group, ID = identification, GFR = glomerular filtration rate, EORTC QLQ-C30 = European Organization for Research and Treatment of Cancer Quality of Life C30 questionnaire, EQ-5D-3L = European Quality of Life Group five dimensions questionnaire 3-level version, ICF = informed consent form, MRI = magnetic resonance imaging, PET = positron emission tomography, PRO = Patient-reported Outcomes.

1 From cycle 3 to the end of treatment, visit on D15 may be scheduled at the discretion of the investigator, if scheduled , visits on D15 should include the same procedures as were performed on D15 C2 (expecting for the plasma biomarker sampling)

a. Screening procedures may be completed during multiple visits but must be completed within specified timeframes before the first dose of study drug..
b. ICF must be signed before any study-related procedures are performed (including screening procedures); this will include consent for tumor tissue collection (primary tumor

and liver metastases) as well as a separate ICF for molecular analyses of whole blood.

c. All CT/MRI scans (chest, abdomen, pelvis, and other suspected sites as applicable) to determine eligibility for randomization must be performed with 4 weeks prior to randomization
d. CT/MRI scans (chest, abdomen, pelvis, and other suspected sites as applicable) will be performed at week 6th and the 12nd week: after CT/MRI scans will be performed at 3-month intervals until disease recurrence as assessed by the investigator.
e. 18FDG-PET CT scan on day 14 (+/-2) after treatment start (results will not be communicated to the patient and the treating physician)
f. ECG, physical examination, and laboratory evaluations are not required on Cycle 1, Day 1 if performed within 7 days of initiation of study drug.
g. After 6 cycles, ECG may be performed at the investigator's discretion. ECG must be performed at End-of treatment.
h. AE assessment is to be started from signing of ICF until 30 days after the last dose of study drug (Safety Follow-up) and at the discretion of the investigator (serious adverse events only) during Active and Survival Follow-up
i. Platelet count (≥100,000 cells/mm3), hemoglobin concentration (≥ 9 g/dL), and absolute neutrophil count (≥1500 cells/mm3) without transfusion or granulocyte-colony stimulating factor and other hematopoietic growth factors must be met at study entry
j. CEC and CEP counts, blood samples to be sored for evaluation of cytokines, circulating angiogenic factors and miRNA plasma levels will be performed on day 14th +/-2 after starting treatment, before starting the 2nd cycle (day 28) at the first restaging (after 6 weeks) and at progression of the disease.
k. Female subjects of childbearing potential must have a negative pregnancy test result ≤ 7 days prior to randomization.
l. A urinalys by dipstick, and a microspic analysis if the urine dipstick tests shows a change from normal
m. GFR > 30mL/min/1.73 m2 according to the Modified Diet in Renal Disease abbreviated formula
n. Subjects who are therapeutically treated with an agent such as warfarin or heparin will be allowed to participate if no underlying abnormality in coagulation parameters exists per medical history. If a subject is taking warfarin or heparin with stable PT-INR at baseline, close monitoring of PT-INR and aPTT should be performed ≥ 1 times/week until PT-INR and aPTT are stable as compared with a pre-dose measurement (defined by the local standard of care)
o. Only if subject is maintained on warfarin or heparin
p. AST, ALT, and bilirubin must be obtained at baseline and monitored weekly during the first 2 cycles of treatment with regorafenib (ie, on Days 1, 8, 15, and 22 of Cycles 1 and 2 [±3 days]), even if values are normal. The investigators may use investigational site or local laboratory AST, ALT, and bilirubin values for subject treatment decisions for Days 8 and 22 of Cycles 1 and 2; these values must be entered in the eCRF.
q. Blood pressure monitoring required weekly for first 6 weeks
r. Subjects discontinuing the study for disease recurrence or any other reason will have a Safety Follow-up Visit 30 (±7 days) after the last dose of study drug and enter Survival Follow-up.
s. Subjects who experience disease recurrence (either during treatment or during Active Follow-up) or otherwise withdraw from the study for any reason other than death will be followed for overall survival unless consent is withdrawn. Such subjects will be evaluated approximately every 3 months to determine their survival status. Survival Follow-up may occur at more frequent intervals at the discretion of the investigator or as per local standard of care. Telephone follow-up is acceptable.

7. RESPONSE EVALUATION

Response must be evaluated through repetition of the CT scan of chest, abdomen and pelvis at week 6th and 12nd from treatment start and thereafter every 3 months.
Response will be codified by the Investigator according to the RECIST (Response Evaluation Criteria In Solid Tumours) guidelines version 1.1, summarised below.

7.1. Measurability of tumour lesions

At the baseline evaluation, the lesions will be defined as follows:

A. **measurable**:	Lesions which can be accurately measured on at least **one dimension** (the longest diameter must be recorded) and found to be ≥10 mm with CT scan or ≥20 mm with conventional techniques.<br><br>Lymph node found to be ≥15 mm in short axis with CT scan.<br><br>Note: All tumor measurements must be recorded in <u>millimeters</u> (or decimal fractions of centimeters).
A. **non-measurable**:	Lesions that are measurable but small (largest diameter <10 mm with CT scan or <20 mm with conventional techniques or pathological lymph nodes with ≥10 to <15 mm short axis).<br><br>Bone lesions, leptomeningeal disease, ascites, pleural/pericardial effusions, lymphangitis of skin or lung, and abdominal masses or organomegaly (that cannot be followed by CT or MRI).<br><br>Lesions that are located in previously irradiates areas.

All measurements must be made using a centimetre rule or gauge.
All the baseline evaluations must be performed as close to the treatment start date as possible, and in all cases no more than 4 weeks earlier. If there is only one measurable lesion, the neoplastic nature of this lesion should be confirmed by cytologic or histologic examination.

7.2. Identification of "target" and "non-target" lesions

All measurable lesions up to a maximum of 2 lesions per organ and 5 lesions in total, representative of all involved organs, should be identified as **target lesions** and recorded and measured at baseline assessment. The target lesions should be selected on the basis of their size (lesions with the longest

diameter), trying to represent all the involved organs. In addition target lesions should be those that lend themselves to reproducible repeated measurements. It may be the case that, on occasion, the largest lesion does not lend itself to reproducible measurement in which circumstance the next largest lesion which can be measured reproducibly should be selected. The sum of the baseline diameters (longest for non-nodal lesions, short axis for nodal lesions) of all target lesions will be calculated, reported in the eCRF, and used as reference for defining the objective response.

All other lesions are identified as **non-target lesions** and must also be recorded at baseline assessment. Measurement of these lesions is not required, but the presence, absence, or the eventual unequivocal progression of each of them must be noted during the follow-up, at each scheduled restaging.

7.3. Evaluation of target lesion response

Complete response (CR):	disappearance of all the target lesions. Any pathological lymph nodes (whether target or non-target) must have reduction in short axis to <10 mm.
Partial Response (PR):	reduction of at least 30% in the sum of diameters of the target lesions, compared to the baseline evaluation.
Progression (P):	increase of at least 20% in the sum of diameters of target lesions and an absolute increase of at least 5 mm, compared to the lowest sum recorded since the start of the treatment, or the appearance of one or more new lesions.
Stable disease (SD):	neither a sufficient reduction to be defined as PR, nor a sufficient increase to be defined as P.

7.4. Evaluation of non target lesion response

Complete Response (CR):	Disappearance of all non target lesions. All lymph nodes must be non-pathological in size (<10 mm short axis)
Non Complete Response / Non Progression (CR/nonP):	Persistence of one or more target lesions
Progression (P):	Appearance of one or more new lesions or indisputable, clear progression of an existing non-target lesion.

7.5. Evaluation of best overall response

The best overall response is the best response recorded from the start of treatment until disease progression/recurrence (taking the lowest measurement recorded since the start of treatment as reference for the progression).

Target Lesions	Non-Target Lesions	New Lesions	**RECIST Response**
CR	CR	No	**CR**
CR	non-CR/non-P	No	**PR**
CR	Not evaluated	No	**PR**
PR	CR o non-CR/non-P or not evaluated	No	**PR**
SD	CR o non-CR/non-P or not evaluated	No	**SD**
Not all evaluated	CR o non-CR/non-P	No	**Not evaluable**
P	Any	Yes or No	**P**
Any	P	Yes or No	**P**
Any	Any	Yes	**P**

An overall deterioration in the state of health leading to a suspension of the treatment without evidence of progression will be defined as a "symptomatic worsening".
Patients who suspend treatment due to symptomatic worsening will be considered as non-responding.

7.6. Reporting of results

All patients included in the study must be assessed for response to treatment, even if they are ineligible. Each patient will be assigned one of the following categories: 1) complete response, 2) partial response, 3) stable disease, 4) progressive disease, 5) early death from malignant disease, 6) early death from toxicity, 7) early death because of other cause, or 8) unknown (not assessable, insufficient data).
All patients will be included in the analysis of the response rate. Patients in response categories 4-8 will be considered as failing to respond to treatment.

8. ADVERSE EVENTS

8.1. Definitions

An **adverse event** is any untoward medical occurrence in a patient or clinical trial subject administered a medicinal product and which does not necessarily have a causal relationship with this treatment.
An **adverse reaction** is an untoward and unintended response to an investigational medicinal product related to any dose administered, judged by either the investigator or the promoter.
An **unexpected adverse reaction** (UAR) is an adverse reaction, the nature or severity of which is not consistent with the applicable product information (e.g. investigator's brochure for an unauthorized investigational product or summary of product characteristics for an authorized product).
A surgical procedure that was planned prior to the start of the study by any physician treating the subject should not be recorded as an AE (however, the condition for which the surgery is required may be an AE).
In the following differentiation between medical history and AEs, the term “condition” may include abnormal physical examination findings, symptoms, diseases, laboratory, or ECG results.

- Conditions that started before signing of informed consent and for which no symptoms or treatment are present until signing of informed consent will be recorded as medical history (eg, seasonal allergy without acute complaints).
- Conditions that started before signing of informed consent and for which symptoms or treatment are present after signing of informed consent, at unchanged intensity, will be recorded as medical history (eg, allergic pollinosis).
- Conditions that started or deteriorated after signing of the ICF will be documented as AEs.

A laboratory test abnormality that is considered to have a clinically relevant effect on the subject (eg, causing the subject to withdraw from the study, requiring treatment or causing apparent clinical manifestations, or judged relevant by the investigator) should be reported as an AE.
A **Suspected Unexpected Serious Adverse Reaction (SUSAR)** is an adverse reaction judged serious by either the investigator and/or the promoter, that is not consistent, either in nature or in severity, with the applicable product information.

8.2. Definition of a serious adverse event

A SAE is classified as any untoward medical occurrence that, at any dose, meets any of the following criteria:

a. Results in death
b. Is life threatening

The term 'life threatening' in the definition refers to an event during which the subject was at risk of death at the time of the event. It does not refer to an event that, hypothetically, might have caused death if it were more severe.

c. Requires in-patient hospitalization or prolongation of existing hospitalization

A hospitalization or prolongation of hospitalization will not be regarded as an SAE if at least one of the following exceptions is met:

- The admission results in a hospital stay of less than 12 hours
- The admission is pre-planned (ie, elective or scheduled surgery arranged prior to the start of the study)
- The admission is not associated with an AE (eg, social hospitalization for purposes of respite care)

However, it should be noted that invasive treatment during any hospitalization may fulfill the criterion of 'medically important' and as such may be reportable as an SAE, dependent on clinical judgment. In addition, where local regulatory authorities specifically require a more stringent definition, the local regulation takes precedence.

d. Results in persistent or significant disability/incapacity

Disability means a substantial disruption of a person's ability to conduct normal life functions.

e. Is a congenital anomaly/birth defect

f. Is another medically important serious event as judged by the investigator

If disease recurrence leads to signs and symptoms that meet the criteria for seriousness (eg, hospitalization), the associated signs and symptoms may be reported as an SAE, but the underlying cause (that is, "recurrent disease" or "metastatic disease") should not be reported as an SAE. In this case, recurrent or metastatic disease should be mentioned on the SAE form as an "alternative explanation."

Additional primary cancers (including skin cancers) regardless of relationship to study treatmen should be reported as SAEs.

An isolated laboratory abnormality that is assigned a severity rating of Grade 4, as defined in NCI-CTCAE v 4.0, is not reportable as an SAE unless the investigator assesses that the event meets standard International Conference on Harmonisation criteria for an SAE. Similarly, a baseline laboratory abnormality that is part of the disease profile should not be reported as an SAE when assigned a severity rating of Grade 4, as defined in NCI-CTCAE v 4.0.

8.3. Collection and reporting of adverse events

All adverse events have to be reported in the toxicity case report form, graded according to the Common Terminology Criteria for Adverse Events (CTCAE) of the National Cancer Institute, version 4.0.

The following safety assessments will be performed at the time points summarized for the study as a whole in Section 6.

The minimum tests required for toxicity evaluation are as follows:

Physical examination

- The physical examination is to include a review of all organ systems, an examination of pertinent organ systems, measurement of vital signs (heart rate, BP, and temperature), and measurement of weight and height (height is to be measured at screening only).
- All study BP measurements should be performed in a consistent manner using the same arm for all evaluations and a manual BP cuff. The subject should sit comfortably for 5 minutes with feet on the floor and the arm supported at heart level prior to assessing BP. Two additional BP measurements, taken 5 minutes apart, should be conducted if the first SBP/DBP reading is > 140/90 mm Hg.

Blood pressure monitoring and Laboratory evaluations

BP values and clinical laboratory evaluations will be performed by a local laboratory at specified time points during the study as described in Table 5.

Table 7: Clinical laboratory evaluations

Laboratory evaluation	Parameters assessed
CBC with differential	RBC count, hemoglobin, hematocrit, platelet count, WBC count with differential counts of neutrophils, lymphocytes, monocytes, basophils, and eosinophils
Electrolytes	sodium, potassium, and chloride
Chemistry	ALT, AST, bilirubin, alkaline phosphatase, triglycerides, uric acid, total protein, albumin, calcium, lipase, phosphate, lactic dehydrogenase, glucose, creatinine, and blood urea nitrogen
Urinalysis via dipstickc	urine appearance, pH, glucose, ketones, erythrocytes, leukocyte esterase, nitrite, bilirubin, urobilinogen, protein, creatinine, protein/creatinine ratio, GFRd
Coagulation panel	PT-INR and aPTTd

a. Specified platelet count (≥ 100,000 cells/mm^3), hemoglobin concentration (≥ 9 g/dL), and absolute neutrophil count (≥ 1500 cells/mm^3) without transfusion, or G-CSF and other hematopoietic growth factors must be met at study entry.

b. ALT, AST, and bilirubin must be obtained at baseline and monitored weekly for the first 2 cycles (ie, on Days 1, 8, 15, and 22 of Cycles 1 and 2 [±3 days]), even if values are normal. The investigators may use investigational site or local laboratory AST, ALT, and bilirubin values for subject treatment decisions; these values must

be entered in the eCRF.

c. Microscopic analysis and proteinuria quantification may be done if the urine dipstick test shows a change from normal.
d. Will be measured according to the MDRD-abbreviated formula. GFR calculations must be done within 7 days of study start, then on Day 1 of the first 6 cycles, and at End-of-treatment.
e. If a subject is taking warfarin or heparin with stable PT-INR at baseline, close monitoring of PT-INR and aPTT should be performed ≥ 1 times/week until PT-INR and aPTT are stable as compared with a pre-dose measurement (defined by the local standard of care).

8.3. Intensity of adverse events

The severity (or intensity) of AEs should be documented using CTCAE v 4.0. If no exact matching code is available in CTCAE v 4.0, the following guide should be used:

- CTC 1 = Mild AE: Transient in nature and generally not interfering with normal activities
- CTC 2 = Moderate AE: Sufficiently discomforting to interfere with normal activities
- CTC 3 = Severe AE: Prevents normal activities
- CTC 4 = Life-threatening and/or disabling AE
- CTC 5 = Results in death (fatal)

8.4. Collection and reporting of serious adverse events (SAE)

All serious adverse events occurring during treatment or until 60 days after surgery must be recorded and reported using the **serious adverse event report form** (SAE form - see enclosures).
The Investigator must immediately report to the sponsor all serious adverse events. The report should be made using the SAE form online or by sending the paper copy by fax (+390817702938) to the coordinating office immediately and not exceeding 24 hours following knowledge of the SAE.
All SAE must be also reported in the toxicity case report form within the corresponding CTCAE term.

8.4.1. Causality assessment between treatment and event

The following criteria will be used for causality assessment:

**Term**	**Description**
CERTAIN	A clinical event, including laboratory test abnormality, occurring in a plausible time relationship to drug administration, and which cannot be explained by concurrent disease or other drugs or chemicals. The response to withdrawal of the drug (dechallenge) should be clinically plausible. The event must be definitive pharmacologically or phenomenologically, using a satisfactory rechallenge procedure if necessary.

PROBABLE/ LIKELY	A clinical event, including laboratory test abnormality, with a reasonable time sequence to administration of the drug, unlikely to be attributed to concurrent disease or other drugs or chemicals, and which follows a clinically reasonable response on withdrawal (dechallenge). Rechallenge information is not required to fulfil this definition.
POSSIBLE	A clinical event, including laboratory test abnormality, with a reasonable time sequence to administration of the drug, but which could also be explained by concurrent disease or other drugs or chemicals. Information on drug withdrawal may be lacking or unclear.
UNLIKELY	A clinical event, including laboratory test abnormality, with a temporal relationship to drug administration which makes a causal relationship improbable, and in which other drugs, chemicals or underlying disease provide plausible explanations.
NOT RELATED	There is no causal relationship between the treatment and the event
CONDITIONAL/ UNCLASSIFIED	A clinical event, including laboratory test abnormality, reported as an adverse reaction, about which more data is essential for a proper assessment or the additional data are under examination.
UNASSESSIBLE/ UNCLASSIFIABLE	A report suggesting an adverse reaction which cannot be judged because information is insufficient or contradictory, and which cannot be supplemented or verified.

8.4.2. Action taken with study treatment

If action is taken with respect to a study drug in order to resolve the SAE, the action is to be documented using the following categories:

- Temporary interruption
- Definitive interruption
- None
- Dose changed
- Unknown

8.4.3. Other specific treatment(s) of serious adverse events

- None
- Remedial drug therapy
- Other

8.4.4. Outcome

The outcome of the SAE is to be documented using the following categories:

- Recovered/resolved
- Recovering/resolving
- Recovered/resolved with sequelae
- Not recovered/not resolved
- Fatal
- Unknown

8.5. Assessments and documentation of adverse events

All AEs occurring from the time the subject has signed the ICF until 30 (±7) days after the last dose of study drug (Safety Follow-up) must be entered in the subject's eCRF and coded according to CTCAE v 4.0.

Documentation of an AE must be supported by an entry in the subject's medical file.

8.6. Expected adverse events

For this study, the applicable reference document is the most current version of the IB.

Overview listings of frequent events that have occurred so far in the clinical development are shown in the current IB. If relevant new safety information is identified, the information will be integrated into an update of the IB and distributed to all participating sites.

The expectedness of AEs will be determined by the Sponsor according to the applicable reference document and according to all local regulations.

8.7. Procedures for safety reporting

The coordinating centre at NCI Naples will:

- review all adverse and serious adverse events reported in the Study and issue queries directly to the Investigator reporting the event
- determine if event qualifies as a SUSAR
- report all SUSARs to the national regulatory authority Agenzia Italiana del Farmaco (AIFA), to all Participating Investigators, and to Ethics Committees of participating centres within the timelines of the article 17 of the European Directive 2001/20/EC.
- provide an annual safety reports, including all Serious Adverse Events occurring in the Study, to all Participating Investigators, to Ethics

Committee of participating centres and to the national regulatory authority Agenzia Italiana del Farmaco (AIFA).

Reporting flow for SAEs and SUSARs

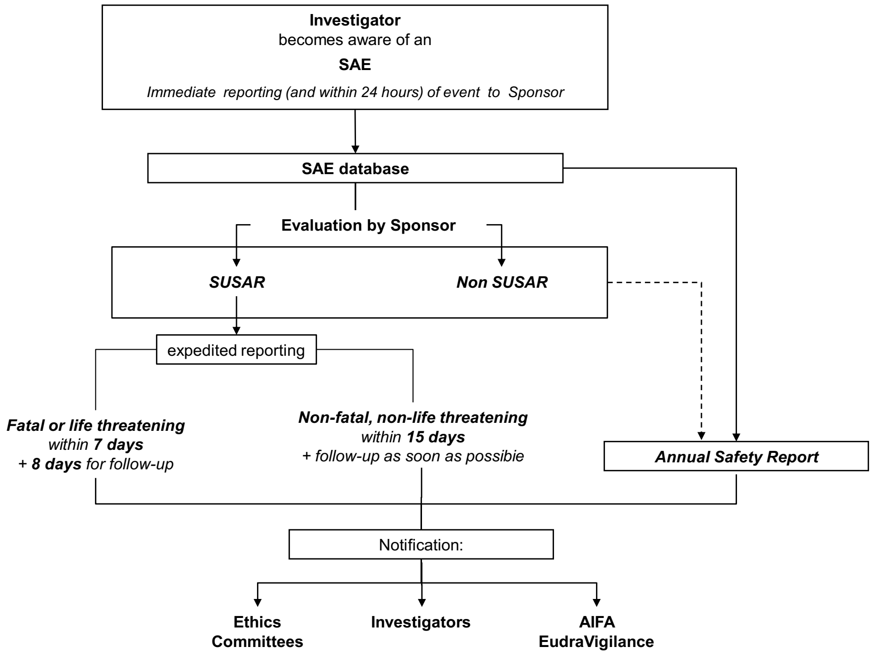

8.8. Pregnancies

The investigator must report to the coordinating centre any pregnancy occurring in a study subject during the subject's participation in this study. The report should be submitted within the same timelines as an SAE, although a pregnancy per se is not considered an SAE.

For a study subject, the outcome of the pregnancy should be followed up carefully, and any outcome of the mother or the child should be reported.

If cases of drug exposure via the father are reported, all efforts should be made to obtain information on course and outcome of the pregnancy, subject to the partner's consent.

For all reports, the forms provided are to be used.

9. STATISTICAL ANALYSIS

All the analyses will be descriptive. Patients will be defined as evaluable if they have assumed at least one regorafenib tablet.

9.1 Primary endpoint

The rate of patients alive and not progressed at 6 months is the primary study end-point. This rate will be calculated by dividing the number of evaluable patients alive and not progressed after 6 months from study entry, by the number of enrolled evaluable patients. Exact 95% confidence intervals will be described.

The following will be considered as events:
- progression of disease at any date within 6 months from registration, according to RECIST criteria (including deterioration of clinical conditions that prevent radiological restaging);
- death for any cause within 6 months from registration;
- interruption of regorafenib due to adverse events followed by initiation of an alternative antineoplastic treatment within 6 months from registration.

If drug-related adverse events lead to definitive termination of regorafenib but the clinical condition of the patient does not require immediate switch to a different antineoplastic treatment, the patient can be followed according to protocol schedule and will be defined as success or failure according to the time of disease progression as above.

9.2. Secondary endpoints

- Toxicity: for each patient and for each type of toxicity, the worst degree suffered during treatment will be described.
- Objective response rate: defined as the number of complete plus partial responses divided by the number of patients enrolled.
- Progression free-survival: defined as the time from registration to progression or death without progression, whichever occurred first. Patients without progression at the end of the study will be censored at the date of the last visit when eventual progression was verified.
- Overall survival: defined as the time from registration to the date of death due to any cause. Patients still alive at the end of the study will be censored on the date of the last information on vital status.
- Metabolic response: defined as a reduction ≥50% of SUVmax after 2 weeks of treatment.

9.3. 1st stage analysis

According the two-stage design, the primary outcome will be evaluated within the first 17 patients and, if needed in order to assess study result, patients enrolment will be temporarily suspended. If 3 or fewer patients will be alive and without progression at 6 months, the study will be stopped early for futility.

10. BIOMARKERS

10.1. Blood sample collection and analysis

Peripheral blood samples will be collected in EDTA tubes through 21G needles and stored as described below:

- at baseline (BL) (4 tubes)
- at day 14 of cycle 1 (C1) (2 tubes)
- at day 28 of cycle 1 (C2) (2 tubes)
- at the first restaging (R) (4 tubes)
- at progression of disease (PD) (4 tubes).

The following tests will be performed on blood samples but, given their uncertain values, these results will not be communicated to the patients and the treating physician:

- **Analysis of polymorphisms** in VEGF, VEGFR, CYP3A4 and UTG1A9 genes (at baseline only).
  Peripheral blood will be collected at BL in EDTA tubes through 21G needles, aliquoted and stored at -80°C. DNA from will be obtained using the DNA extraction kit (Qiagen/SABiosciences) following manufacturer instructions. The expression of the indicated polymorphisms will be evaluated by using pyrosequencing technology with specific 5'-biotinylated primers. Sequencing primers will be developed using SNP Primer Design software from Biotage. Genotyping will be done using a PyroMark ID pyrosequencer according to the manufactor's instructions. Polymorphisms of these genes may affect activity and toxicity of treatment. One peripheral blood sample for each patient is adequate.

- **Analysis of CECs and CEPs** (at each of the above reported time-points).
  Peripheral blood will be collected, at all the time points previously described, in EDTA tubes through 21G needles and cell suspensions will be evaluated, after red cell lyses. Analysis of CEC/CEP should be performed within 24 h from collection of sample stored at 4 °C by flow cytometry. CECs are defined as negative for haematopoietic marker CD45, positive for endothelial markers P1H12. CEPs are defined as negative for haematopoietic marker CD45, positive for endothelial markers VEGF-R2 and positive for progenitor marker CD34.Vitality will be evaluated by staining with Syto 16.

- **Analysis of circulating angiogenic factors and cytokines** (at each of the above reported time-points).
  Peripheral blood will be collected in EDTA tubes through 21G needles aliquoted and stored at -80°C. The analysis will be done with the Bio-Plex^{TM} thecnology.

- **Analysis of cfDNA mutations** (at each of the above reported time-points).
  Peripheral blood will be collected at BL in EDTA tubes through 21G needles, aliquoted and stored at -80°C. After Genome equivalent absolute quantification, samples will be processed using the *QIAamp Circulating*

Nucleic acid Kit (Qiagen/SABiosciences) according to manufacturer' instructionse and mutation analysisis will be performed by BEAMing, Droplet digital PCR analysis or Next Generation Sequencing analysis.

- **Analysis of miRNA** (<u>at each of the above reported time-points</u>). Peripheral blood will be collected at BL in EDTA tubes through 21G needles, aliquoted and stored at -80°C. Samples will be processed using the *miRNeasy Mini kit* (Qiagen/SABiosciences) e enriched using the *RT2 qPCR-Grade miRNA Isolation Kit*, *MA-01* (Qiagen /SABiosciences). After miRNA retrotranscription as cDNA by the *RT² miRNA First Strand Kit* (Qiagen/SABiosciences), miRNA expression will be measured using the *Human Serum RT² miRNA PCR Array* (Qiagen/SABiosciences) that contains 85 sequences normally found in human serum and fluids, including those found altered in cancer patients.

10.2. Statistical analysis of biomarkers

Due to the small sample size, statistical analysis of biomarkers data will be conducted with the aim of hypothesis generation.
First of all, a complete description of data from biological and pharmacogenomic studies will be done. For biomarkers that might change over time as a consequence of treatment, levels before and after treatment will be compared with appropriate statistical tests, based on the type of data.
Outcomes of treatment will be compared between different VEGF and VEGFR phenotypes, with appropriate statistical tests.
P values ≤0.05 will be considered significant, and no adjustment is planned for multiple comparisons due to the exploratory nature of the analysis.

11. TUMOR METABOLIC EVALUATION

Our group has shown that early FDG-PET-CT is able to predict survival outcomes in colorectal rectal cancer (32). Therefore PET-CT scans are planned at:

- baseline (within 2 weeks before treatment start)
- 14 (+/- 2) days after treatment start.

Patients will be fasted for at least 6 hours and blood glucose level will have to be less than 150 mg/dL for the study to be eligible for further quantitative analysis.
PET images will be reconstructed using iterative reconstruction and normalized for injected dose and patient body weight. Image analysis will be performed utilizing a semi-automatic region-of-interest (ROI) drawing software package where a three-dimensional region will be drawn around the area of increased uptake. Threshold values will be adjusted in order to encompass the area of increased uptake visually.

For each tumor volume, the following parametres will be calculated:

- SUV = (measured activity concentration [Bq/mL])/(injected activity [Bq]/body weight [kg] · 1,000).
    - SUV-max = the maximum pixel value measured in the visualized lesion
    - SUV-mean = the average activity values in the ROIs.
- TLG (Total Lesion Glycolysis) = SUV-mean x metabolic tumor volume (mm^3)

On the basis of these parameters, the following indicator will be calculated for each patients, in order to assess metabolic response:

- Highest SUVmax = the highest SUVmax value among all the evaluable lesions
- Total SUVmax = the sum of the SUVmax value of all the evaluable lesions
- Highest TLG = the highest TLG value among all the evaluable lesions
- Total TLG = the sum of the TLG value of all the evaluable lesions

PET response will be calculated by measuring changes reported at the post treatment examination (14 +/- 2 days after treatment start) compared to the baseline test. For all the indicators the change will be calculated as:

Δ = (value-post – value-baseline)/value-baseline x 100

Consistent with previous studies (32-34) the threshold to define a patient as responder according to change of SUV or TLG indicators is ≤ -50%. Therefore, patients with any change above these thresholds will be defined as non-responder. This value will be validated within this study for its predictive ability of survival outcome. Further thresholds will be eventually explored only in case of failure (lack of predictive ability) of the proposed validation.

12. QUALITY ASSURANCE AND MONITORING

The procedures set out in this study protocol are designed to ensure that the principles of the Good Clinical Practice guidelines of the International Conference on Harmonization (ICH) and the Declaration of Helsinki are respected in the conduct, evaluation and documentation of this study.
Centralized monitoring activities are planned for 100% of the data. Peripheral monitoring of source data will be performed at discretion of the coordinating centre. , with peripheral auditing visits planned in case of need.

13. DATA COLLECTION PROCEDURES

Patient registration and data collection are centralized at the Clinical Trials Unit of the National Cancer Institute of Naples.

Patient registration is web-based (http://www.usc-intnapoli.net) or by telephone.
Data collection is electronic at the above website.

13.1. Contacts

Clinical Trials Unit
National Cancer Institute Naples
tel: + 39 081-5903571
fax: + 39 081-7702938

For registration and data collection:
Manuela Florio, Giovanni de Matteis, Giuliana Canzanella, Federika Crudele, Cristiana De Luca
datamanager@usc-intnapoli.net

For Ethical Committees and Administration
Marilena Martino, Teresa Ribecco
marilena.martino@usc-intnapoli.net, teresa.ribecco@usc-intnapoli.net

For drug management and pharmacovigilance
Antonia Del Giudice
antonia.delgiudice@usc-intnapoli.net

14. PERSONAL DATA PROTECTION PROCEDURES

In order to protect the *privacy* of patients enrolled in the study, the following procedures will be used:

- the Coordinating Center, which has centralized responsibility for registration, randomization and data collection and processing procedures, will not allow access to the data, except to public authorities responsible for inspections under the relevant legislation (Health Ministry, Ethics Committee);
- at registration, patients will be assigned a numerical identification code (corresponding to progressive numbers of patients enrolled in the study) that will be noted together with the participating center's code for all the CRFs (web or paper based) and that will be used in all communications regarding individual patients;
- a list of patient codes and names will be kept by the Participating Investigator Center;
- documents that are not for submission to the Coordinating Center (eg, signed informed consent forms) should be kept in strict confidence by the investigator.

15. ETHICAL CONSIDERATIONS

15.1. Risk of undertreatment

The Simon's optimal two-stage study design has been chosen in order to minimize the number of patients exposed to a potentially inactive treatment, considering that alternative treatment options exist, although not satisfactory and likely to be more toxic. Moreover, an intensive schedule of response evaluation has been planned (6 and 12 weeks after treatment start) that allows to detect cases of early progression, with the aim of offering patients alternative treatment options.

15.2. Risk of toxicity (overtreatment)

In principle, there is no risk of overtreatment as this study is evaluating the activity of a biologic drug monotherapy in a population that alternatively may receive in clinical practice a combination chemotherapy that is expected to be more toxic. Moreover, an intensive clinical and laboratory assessment is scheduled for the first two cycles of treatment, to closely monitor the occurrence of regorafenib-related adverse events (that are generally early and temporary).

16. SUBJECTS INFORMATION AND CONSENT

All relevant information on the study will be summarized in an integrated subject information sheet and ICF provided by the Sponsor or the study center. A sample subject information and ICF is provided as a document separate to this protocol.

Based on this subject information sheet, the investigator or designee will explain all relevant aspects of the study to each subject/legal representative or proxy consenter (if the subject is under legal protection), prior to his/her entry into the study (ie, before any examinations and procedures associated with the selection for the study are performed or any study-specific data are recorded on study-specific forms).

The investigator will also mention that written approval of the IEC/IRB has been obtained.

Each subject/legal representative or proxy consenter will have ample time and opportunity to ask questions and will be informed about the right to withdraw from the study at any time without any disadvantage and without having to provide reasons for this decision.

Only if the subject/legal representative or proxy consenter voluntarily agrees to sign the ICF and has done so, may the subject enter the study. Additionally, the investigator and other information provider (if any) will personally sign and date the form. The subject/legal representative or proxy consenter will receive a copy of the signed and dated form.

The signed ICF is to remain in the investigator site file or, if locally required, in the subject's note/file of the medical institution.

The ICF and any other written information provided to subjects/legal representatives or proxy consenters will be revised whenever important new information becomes available that may be relevant to the subject's consent, or there is an amendment to the protocol that necessitates a change to the content of the subject information and/or the written ICF. The investigator will inform the subject/legal representative or proxy consenter of changes in a timely manner and will ask the subject to confirm his/her participation in the study by signing the revised ICF. Any revised written informed consent form and written information must receive the IEC's/IRB's approval/favorable opinion in advance of use.

17. ADMINISTRATIVE ASPECTS

- The study is a non-profit investigator initiated trial.
- The National Cancer Institute of Napoli is the non-profit sponsor of the study and will be responsible for protocol development, regulatory approvals, data collection, drug distribution and pharmacovigilance, monitoring, data analysis and publication.
- Regorafenib will be provided for free by the manufacturer.
- The study is planned to be multicentre in Italy and 5 to 10 centres are expected to participate.
- The sponsor will provide an insurance policy to cover possible damages caused to patients participating in the trial. The policy will cover all participating centers.
- Study protocol, patient information and informed consent will be submitted to Independent Ethical Committees and will only be started after their approval. Independent Ethical Committees will be periodically informed of study progress, safety and planned or premature end of the study.

18. REFERENCES

1. Ferlay J, Steliarova-Foucher E, Lortet-Tieulent J, Rosso S, Coebergh JW, Comber H, et al. Cancer incidence and mortality patterns in Europe: estimates for 40 countries in 2012. European journal of cancer. 2013 Apr;49(6):1374-403. PubMed PMID: 23485231.
2. Efficacy of intravenous continuous infusion of fluorouracil compared with bolus administration in advanced colorectal cancer. Meta-analysis Group In Cancer. Journal of clinical oncology : official journal of the American Society of Clinical Oncology. 1998 Jan;16(1):301-8. PubMed PMID: 9440757.
3. Grothey A, Sargent D, Goldberg RM, Schmoll HJ. Survival of patients with advanced colorectal cancer improves with the availability of fluorouracil-leucovorin, irinotecan, and oxaliplatin in the course of treatment. Journal of clinical oncology : official journal of the American Society of Clinical Oncology. 2004 Apr 1;22(7):1209-14. PubMed PMID: 15051767.
4. de Gramont A, Figer A, Seymour M, Homerin M, Hmissi A, Cassidy J, et al. Leucovorin and fluorouracil with or without oxaliplatin as first-line treatment in advanced colorectal cancer. Journal of clinical oncology : official journal of the American Society of Clinical Oncology. 2000 Aug;18(16):2938-47. PubMed PMID: 10944126.
5. Saltz LB, Clarke S, Diaz-Rubio E, Scheithauer W, Figer A, Wong R, et al. Bevacizumab in combination with oxaliplatin-based chemotherapy as first-line therapy in metastatic colorectal cancer: a randomized phase III study. Journal of clinical oncology : official journal of the American Society of Clinical Oncology. 2008 Apr 20;26(12):2013-9. PubMed PMID: 18421054.
6. Van Cutsem E, Kohne CH, Hitre E, Zaluski J, Chang Chien CR, Makhson A, et al. Cetuximab and chemotherapy as initial treatment for metastatic colorectal cancer. The New England journal of medicine. 2009 Apr 2;360(14):1408-17. PubMed PMID: 19339720.
7. Douillard JY, Siena S, Cassidy J, Tabernero J, Burkes R, Barugel M, et al. Randomized, phase III trial of panitumumab with infusional fluorouracil, leucovorin, and oxaliplatin (FOLFOX4) versus FOLFOX4 alone as first-line treatment in patients with previously untreated metastatic colorectal cancer: the PRIME study. Journal of clinical oncology : official journal of the American Society of Clinical Oncology. 2010 Nov 1;28(31):4697-705. PubMed PMID: 20921465.
8. Kopetz S, Chang GJ, Overman MJ, Eng C, Sargent DJ, Larson DW, et al. Improved survival in metastatic colorectal cancer is associated with adoption of hepatic resection and improved chemotherapy. Journal of clinical oncology : official journal of the American Society of Clinical Oncology. 2009 Aug 1;27(22):3677-83. PubMed PMID: 19470929. Pubmed Central PMCID: 2720081.
9. Tournigand C, Andre T, Achille E, Lledo G, Flesh M, Mery-Mignard D, et al. FOLFIRI followed by FOLFOX6 or the reverse sequence in advanced colorectal cancer: a randomized GERCOR study. Journal of clinical oncology : official journal of the American Society of Clinical Oncology. 2004 Jan 15;22(2):229-37. PubMed PMID: 14657227.
10. Karapetis CS, Khambata-Ford S, Jonker DJ, O'Callaghan CJ, Tu D, Tebbutt NC, et al. K-ras mutations and benefit from cetuximab in advanced

colorectal cancer. The New England journal of medicine. 2008 Oct 23;359(17):1757-65. PubMed PMID: 18946061.

11. Amado RG, Wolf M, Peeters M, Van Cutsem E, Siena S, Freeman DJ, et al. Wild-type KRAS is required for panitumumab efficacy in patients with metastatic colorectal cancer. Journal of clinical oncology : official journal of the American Society of Clinical Oncology. 2008 Apr 1;26(10):1626-34. PubMed PMID: 18316791.
12. Douillard JY, Oliner KS, Siena S, Tabernero J, Burkes R, Barugel M, et al. Panitumumab-FOLFOX4 treatment and RAS mutations in colorectal cancer. The New England journal of medicine. 2013 Sep 12;369(11):1023-34. PubMed PMID: 24024839.
13. Van Cutsem E, Tabernero J, Lakomy R, Prenen H, Prausova J, Macarulla T, et al. Addition of aflibercept to fluorouracil, leucovorin, and irinotecan improves survival in a phase III randomized trial in patients with metastatic colorectal cancer previously treated with an oxaliplatin-based regimen. Journal of clinical oncology : official journal of the American Society of Clinical Oncology. 2012 Oct 1;30(28):3499-506. PubMed PMID: 22949147.
14. Bennouna J, Sastre J, Arnold D, Osterlund P, Greil R, Van Cutsem E, et al. Continuation of bevacizumab after first progression in metastatic colorectal cancer (ML18147): a randomised phase 3 trial. The lancet oncology. 2013 Jan;14(1):29-37. PubMed PMID: 23168366.
15. Kubicka S, Greil R, Andre T, Bennouna J, Sastre J, Van Cutsem E, et al. Bevacizumab plus chemotherapy continued beyond first progression in patients with metastatic colorectal cancer previously treated with bevacizumab plus chemotherapy: ML18147 study KRAS subgroup findings. Annals of oncology : official journal of the European Society for Medical Oncology / ESMO. 2013 Sep;24(9):2342-9. PubMed PMID: 23852309.
16. Wilhelm SM, Dumas J, Adnane L, Lynch M, Carter CA, Schutz G, et al. Regorafenib (BAY 73-4506): a new oral multikinase inhibitor of angiogenic, stromal and oncogenic receptor tyrosine kinases with potent preclinical antitumor activity. International journal of cancer Journal international du cancer. 2011 Jul 1;129(1):245-55. PubMed PMID: 21170960.
17. Grothey A, Van Cutsem E, Sobrero A, Siena S, Falcone A, Ychou M, et al. Regorafenib monotherapy for previously treated metastatic colorectal cancer (CORRECT): an international, multicentre, randomised, placebo-controlled, phase 3 trial. Lancet. 2013 Jan 26;381(9863):303-12. PubMed PMID: 23177514.
18. Duda DG, Cohen KS, di Tomaso E, Au P, Klein RJ, Scadden DT, et al. Differential CD146 expression on circulating versus tissue endothelial cells in rectal cancer patients: implications for circulating endothelial and progenitor cells as biomarkers for antiangiogenic therapy. Journal of clinical oncology : official journal of the American Society of Clinical Oncology. 2006 Mar 20;24(9):1449-53. PubMed PMID: 16549839. Pubmed Central PMCID: 2718681.
19. Ronzoni M, Manzoni M, Mariucci S, Loupakis F, Brugnatelli S, Bencardino K, et al. Circulating endothelial cells and endothelial progenitors as predictive markers of clinical response to bevacizumab-based first-line treatment in advanced colorectal cancer patients. Annals of oncology : official

journal of the European Society for Medical Oncology / ESMO. 2010 Dec;21(12):2382-9. PubMed PMID: 20497963.
20. Malka D, Boige V, Jacques N, Vimond N, Adenis A, Boucher E, et al. Clinical value of circulating endothelial cell levels in metastatic colorectal cancer patients treated with first-line chemotherapy and bevacizumab. Annals of oncology : official journal of the European Society for Medical Oncology / ESMO. 2012 Apr;23(4):919-27. PubMed PMID: 21825101.
21. Matsusaka S, Suenaga M, Mishima Y, Takagi K, Terui Y, Mizunuma N, et al. Circulating endothelial cells predict for response to bevacizumab-based chemotherapy in metastatic colorectal cancer. Cancer chemotherapy and pharmacology. 2011 Sep;68(3):763-8. PubMed PMID: 21170650.
22. Calleri A, Bono A, Bagnardi V, Quarna J, Mancuso P, Rabascio C, et al. Predictive Potential of Angiogenic Growth Factors and Circulating Endothelial Cells in Breast Cancer Patients Receiving Metronomic Chemotherapy Plus Bevacizumab. Clinical cancer research : an official journal of the American Association for Cancer Research. 2009 Dec 15;15(24):7652-7. PubMed PMID: 19996223.
23. Goon PK, Lip GY, Boos CJ, Stonelake PS, Blann AD. Circulating endothelial cells, endothelial progenitor cells, and endothelial microparticles in cancer. Neoplasia. 2006 Feb;8(2):79-88. PubMed PMID: 16611400. Pubmed Central PMCID: 1578513.
24. Asahara T, Murohara T, Sullivan A, Silver M, van der Zee R, Li T, et al. Isolation of putative progenitor endothelial cells for angiogenesis. Science. 1997 Feb 14;275(5302):964-7. PubMed PMID: 9020076.
25. Willett CG, Boucher Y, di Tomaso E, Duda DG, Munn LL, Tong RT, et al. Direct evidence that the VEGF-specific antibody bevacizumab has antivascular effects in human rectal cancer. Nature medicine. 2004 Feb;10(2):145-7. PubMed PMID: 14745444. Pubmed Central PMCID: 2693485.
26. Willett CG, Duda DG, di Tomaso E, Boucher Y, Ancukiewicz M, Sahani DV, et al. Efficacy, safety, and biomarkers of neoadjuvant bevacizumab, radiation therapy, and fluorouracil in rectal cancer: a multidisciplinary phase II study. Journal of clinical oncology : official journal of the American Society of Clinical Oncology. 2009 Jun 20;27(18):3020-6. PubMed PMID: 19470921. Pubmed Central PMCID: 2702234.
27. Jain L, Vargo CA, Danesi R, Sissung TM, Price DK, Venzon D, et al. The role of vascular endothelial growth factor SNPs as predictive and prognostic markers for major solid tumors. Molecular cancer therapeutics. 2009 Sep;8(9):2496-508. PubMed PMID: 19755511. Pubmed Central PMCID: 2751847.
28. Elens L, Nieuweboer A, Clarke SJ, Charles KA, de Graan AJ, Haufroid V, et al. CYP3A4 intron 6 C>T SNP (CYP3A4*22) encodes lower CYP3A4 activity in cancer patients, as measured with probes midazolam and erythromycin. Pharmacogenomics. 2013 Jan;14(2):137-49. PubMed PMID: 23327575.
29. Peeters M, Price TJ, Cervantes A, Sobrero AF, Ducreux M, Hotko Y, et al. Final results from a randomized phase 3 study of FOLFIRI {+/-} panitumumab for second-line treatment of metastatic colorectal cancer. Annals of oncology : official journal of the European Society for Medical Oncology / ESMO. 2014 Jan;25(1):107-16. PubMed PMID: 24356622.

30. Peeters M, Price TJ, Cervantes A, Sobrero AF, Ducreux M, Hotko Y, et al. Randomized phase III study of panitumumab with fluorouracil, leucovorin, and irinotecan (FOLFIRI) compared with FOLFIRI alone as second-line treatment in patients with metastatic colorectal cancer. Journal of clinical oncology : official journal of the American Society of Clinical Oncology. 2010 Nov 1;28(31):4706-13. PubMed PMID: 20921462.
31. Cohn AL, Tabernero J, Maurel J, Nowara E, Sastre J, Chuah BY, et al. A randomized, placebo-controlled phase 2 study of ganitumab or conatumumab in combination with FOLFIRI for second-line treatment of mutant KRAS metastatic colorectal cancer. Annals of oncology : official journal of the European Society for Medical Oncology / ESMO. 2013 Jul;24(7):1777-85. PubMed PMID: 23510984.
32. Lastoria S, Piccirillo MC, Caraco C, Nasti G, Aloj L, Arrichiello C, et al. Early PET/CT scan is more effective than RECIST in predicting outcome of patients with liver metastases from colorectal cancer treated with preoperative chemotherapy plus bevacizumab. Journal of nuclear medicine : official publication, Society of Nuclear Medicine. 2013 Dec;54(12):2062-9. PubMed PMID: 24136935.
33. Avallone A, Aloj L, Caraco C, Delrio P, Pecori B, Tatangelo F, et al. Early FDG PET response assessment of preoperative radiochemotherapy in locally advanced rectal cancer: correlation with long-term outcome. European journal of nuclear medicine and molecular imaging. 2012 Dec;39(12):1848-57. PubMed PMID: 23053320.
34. Cascini GL, Avallone A, Delrio P, Guida C, Tatangelo F, Marone P, et al. 18F-FDG PET is an early predictor of pathologic tumor response to preoperative radiochemotherapy in locally advanced rectal cancer. Journal of nuclear medicine : official publication, Society of Nuclear Medicine. 2006 Aug;47(8):1241-8. PubMed PMID: 16883000.

19. APPENDICES

19.1. Eastern Cooperative Oncology Group performance status

Grade	Description
0	Fully active, able to carry on all pre-disease performance without restriction
1	Restricted in physically strenuous activity but ambulatory and able to carry out work of a light or sedentary nature (eg, light housework, office work)
2	Ambulatory and capable of all self-care but unable to carry out any work activities. Up and about more than 50% of waking hours.
3	Capable of only limited self-care, confined to bed or chair more than 50% of waking hours
4	Completely disabled. Cannot carry on any self-care. Totally confined to bed or chair.
5	Death

19.2. Glomerular filtration rate

In accordance with established nephrology practice and guidelines, renal function at baseline and throughout the study will be assessed by means of the estimated GFR, calculated using the abbreviated MDRD study formula.
This equation of four variables (serum creatinine level, age, sex, and ethnicity) is recommended by the National Kidney Foundation for use in individuals 18 years or older. The formula is as follows:
aMDRD formula
GFR (mL / min / 1.73m^2) = k x 186 x [serum creatinine]$^{-1.154}$ x [age]$^{-0.203}$
Where k = 1 (men) or 0.742 (women), GFR indicates glomerular filtration rate, and serum creatinine is measured in mg/dL.
Correction factor for eGFR calculation is 0.881 for Japanese subjects, 1.227 for Chinese subjects, and 1.110 for Korean subjects.[a]
Subjects with a baseline GFR < 30 mL/min calculated by this method will not be allowed to participate in the study.

a. Matsuo S, Yasuda Y, Imai E, Horio, M (2010): Current status of estimated glomerular filtration rate (eGFR) equations for Asians and an approach to create a common eGFR equation. Nephrology 15, 45-48.

19.3. CYP3A4 inhibitors/inducers

The strong inhibitors and inducers of CYP3A4 listed here should be avoided during this study:

CYP 3A4 Inhibitors	CYP 3A4 Inducers
Amiodarone	Ajmaline [d]
Aprepitant [a]	Avasimibe [d]
Boceprevir [c]	Barbiturates
Chloramphenicol	Carbamazepine [d]
Cimetidine [b]	Enzalutaminde [d]
Ciprofloxacin	Efavirenz
Clarithromycin [c]	Fosphenytoin [d]
Cobicistat [c]	Glucocorticoids
Delaviridine [c]	Methylphenobarbital [d]
Diethyl-Dithiocarbamate	Mitotane [d]
Diltiazem [a]	Modafinil
Erythromycin [a]	Nevirapine
Fluconazole [a]	Oxcarbazepine
Fluvoxamine	Phenobarbital [d]
Gestodene	Phenytoin [d]
Grapefruit Juice [c]	Pioglitazone
Imatinib	Primidone [d]
Indinavir [c]	Rifabutin
Itraconazole [c]	Rifampicin [d]
Ketoconazole [c]	Rifampin [d]
Lopinavir [c]	Rifamycin [d]
Mibefradil [c]	Rifapentin [d]
Miconazole [c]	St. John's Wort [d]
Mifepristone	Troglitazone
Nefazodone [c]	
Nelfinavir [c]	
Norfloxacin	
Norfluoxetine	
Posaconazole [c]	
Ritonavir [c]	
Saquinavir [c]	
Starfruit	
Telaprevir [c]	
Telithromycin [c]	
Tipranavir [c]	
Troleandomycin [c]	
Verapamil [a]	
Voriconazole [c]	

a. A moderate inhibitor is one that causes a > 2-fold increase in the plasma AUC values or 50-80% decrease in clearance.
b. A weak inhibitor is one that causes a > 1.25-fold but < 2-fold increase in the plasma AUC values or 20-50% decrease in clearance.
c. A strong inhibitor is one that causes a > 5-fold increase in the plasma AUC values or more than 80% decrease in clearance, and are NOT allowed during this clinical trial.
d. Strong inducers are not allowed during this clinical trial.

Source: Prescribing Information for Regorafenib (SEP 2012) and Indiana University School of Medicine, Division of Clinical Pharmacology (Update: 25 JAN 2012): http://medicne.iupui.edu/clinpharm/ddis/table.aspx

19.4. Common Terminology Criteria for Adverse Events

This study will utilize the CTCAE Version 4.0 for toxicity and serious adverse event reporting. A copy of the CTCAE Version 4.0 can be downloaded in PDF form from http://evs.nci.hin.gov/ftp1/CTCAE/About.html.
All appropriate treatment areas should have access to a copy of the CTCAE Version 4.0.

19.5. New York Heart Association functional classification

Class	NYHA Functional Classification
I	Patients have cardiac disease but *without* the resulting *limitations* of physical activity. Ordinary physical activity does not cause undue fatigue, palpitation, dyspnea, or anginal pain.
II	Patients have cardiac disease resulting in *slight limitation* of physical activity. They are comfortable at rest. Ordinary physical activity results in fatigue, palpitation, dyspnea, or anginal pain.
III	Patients have cardiac disease resulting in *marked limitation* of physical activity. They are comfortable at rest. Less than ordinary physical activity causes fatigue, palpitation, dyspnea, or anginal pain.
IV	Patients have cardiac disease resulting in *inability* to carry on any physical activity without discomfort. Symptoms of cardiac insufficiency or of the anginal syndrome may be present even at rest. If any physical activity is undertaken, discomfort is increased.

19.6. Examples of a low-fat breakfast

Two slices of white toast with 1 tablespoon of low-fat margarine and 1 tablespoon of jelly, and 250 ml of skim milk (approximately 319 calories and 8.2 grams of fat)

One cup of cereal, 250 ml of skimmed milk, 1 piece of toast with jam (no butter or marmalade), apple juice, and 1 cup of coffee or tea (2 grams fat, 17 grams protein, 93 grams carbohydrate; 520 calories).

Note: regorafenib should not be taken with grapefruit juice.

Per il Promotore

Dr.ssa Maria Carmela Piccirillo

Firma ________________________ Data____/____/________
